# Avian community characteristics and demographics reveal how conservation value of regenerating tropical dry forest changes with forest age

**DOI:** 10.7717/peerj.5217

**Published:** 2018-07-10

**Authors:** Steven C. Latta, Nathan L. Brouwer, Danilo A. Mejía, Maria M. Paulino

**Affiliations:** 1 Department of Conservation and Field Research, National Aviary, Pittsburgh, PA, United States of America; 2 Grupo Acción Ecológica, La Joya, Dominican Republic

**Keywords:** Buffer zones, Agroecosystems, Habitat change, Avian abundance, Land use, Neotropical migratory birds, Chronosequence, Endemics, Landscape matrix, Hispaniola

## Abstract

Expansion of secondary forests following the abandonment of agriculture may have important implications for bird conservation, but few studies have examined the dynamics of this process. We studied bird use of a chronosequence of differently-aged abandoned pastures regenerating to dry forest to better understand how the value of these habitats to birds changes over time. In a five year study on Hispaniola, we recorded 7,315 net captures of 60 species of landbirds in sites that began the study at two, five, 10, and 20 years post-abandonment, and in mature native dry forest. Twenty-five species made up 97% of all net captures. Highest capture rates were in the two youngest sites. These early-successional habitats had many over-wintering Neotropical migrants; among residents, granivores and frugivores predominated. In contrast, both the twenty-year-old and mature forest sites had few migrants, more resident insectivores and omnivorous species, and a greater proportion of endemics. Age and sex ratios, body condition and site persistence suggest early successional sites were sub-optimal for most over-wintering migrants, but habitat improved with age for three migratory species; results for permanent residents varied among species. Remnant trees and understory shrubs in the agroecological matrix likely contributed to avian diversity in regenerating dry forest sites, and proximity to mature forest also likely affected the diversity and abundance of birds in regenerating habitat. Our study shows that regenerating forests do not fully compensate for loss of mature dry forest habitat, even after 24 years of regeneration; natural restoration of complex microhabitats in dry forest sites converted to agriculture may take decades or longer. The highest value of regenerating forests may be as habitat for some over-wintering Neotropical migrants, and in creating a buffer zone that enhances biodiversity conservation by re-integrating these lands into the protected tracts of mature forest needed by the islands more unique and endemic bird species.

## Introduction

Human activity has directly affected half of the earth’s ice-free land surface, with approximately 40% of land now dedicated to agricultural crops or pasture (GEF, 2012). The resulting expansion of degraded and deforested lands has important implications for long-term conservation of wildlife (Daily, Ehrlich & Sanchez-Azofeifa, 2001). But increasingly, attention is being paid to maximizing the conservation value of agricultural lands (Vandermeer & Perfecto, 1997; Petit & Petit, 2003), with investigations seeking to understand how birds respond to changes in land use in the agricultural matrix surrounding mature forest (Daily, Ehrlich & Sanchez-Azofeifa, 2001; Hughes, Daily & Ehrlich, 2002; Sekercioglu et al., 2002). Several studies have shown that tropical forest birds are not entirely isolated from the agricultural matrix (Greenberg, Bichier & Sterling, 1997; Daily, Ehrlich & Sanchez-Azofeifa, 2001; Sekercioglu et al., 2007), and that this matrix may provide an important buffer between forested reserves and other, more intensive anthropogenic land uses (Lynagh & Urich, 2002). The conservation of biodiversity, and even the functioning of national parks and reserves as repositories of species diversity, is thus increasingly seen to be dependent upon how we manage the surrounding agricultural landscapes (Vandermeer & Perfecto, 1997; Daily, Ehrlich & Sanchez-Azofeifa, 2001; Green et al., 2005; Sekercioglu et al., 2007).

As a component of the agricultural matrix, pastures are one of the most abundant agricultural habitats worldwide, but the potential conservation value of pastures has been generally overlooked (Harvey et al., 2006). Abandoned pastures also represent one of our best opportunities for restoration of forests, especially when some remnant tree cover has been retained for shade, live fences, or other purposes. Shifting agricultural priorities and economic markets, as well as widespread slash-and-burn agricultural practices, can result in local or landscape-scale abandonment of pastures, resulting in a patchwork of pastures, scrub and early-successional regeneration within the agricultural matrix (Harvey et al., 2006). Studies have documented presence/absence or behavioral attributes of birds in scrub or agricultural habitats (Johnson & Sherry, 2001; Komar, 2006), but the employment of avian survival or other demographic variables in these studies is seldom realized despite its importance in assessing habitat quality for birds (but see Wunderle & Latta, 2000; Johnson et al., 2006).

Pastures are also one of the most common land use types on the island of Hispaniola (Tolentino & Peña, 1998). The island’s two nations, Haiti and the Dominican Republic, harbor one of the most diverse assemblages of birds in the Caribbean, with more endemic bird species than any other Caribbean island. Its contribution to global biodiversity has earned Hispaniola the highest ranking of importance in a worldwide assessment of bird protection priorities (Stattersfield et al., 1998). Over-wintering Neotropical migratory birds are also an important seasonal component of the avifauna (Latta et al., 2006). Forest habitats are vital to the survival of many endemic and migrant bird species, but the loss of these habitats on Hispaniola has been estimated as >90% (Stattersfield et al., 1998). In response to this crisis, the Dominican government has established a number of protected areas. But the value of regenerating pastures and other agricultural lands, or how they can be managed or restored for birds, has received almost no attention.

The primary objectives of this research were to: (1) determine the abundance and diversity of birds across a chronosequence of different-aged dry forest regenerating from pastures; (2) determine how endemics, permanent residents, over-wintering migratory birds, and birds representing different foraging guilds respond to these regenerating dry forest habitats; (3) determine if there are differences in demographic structure or site persistence among birds occurring in these same early-successional habitats; and (4) compare these results to similar data from mature dry forests representing the native habitat of these sites prior to their deforestation. Based on these findings, we discuss the conservation value of regenerating dry forest in an agroecological matrix.

## Methods

### Study sites

We studied bird communities during the northern winters of 2003–04 through 2007–08 in the buffer zone of Sierra de Bahoruco National Park, Dominican Republic where a growing human population and associated agricultural activities often conflict with park protection goals (Pasachnik, Carreras De León & León, 2016). Four study sites of >15 ha were established at elevations of ∼400 m within 2 km of the village of Mencia (18.176°, −71.737°). Sites were chosen based on age since disturbance (confirmed through interviews with landowners and long-term residents). The sites were originally cleared for agriculture (primarily beans, squash), and then utilized for occasional low-density grazing by small numbers of livestock. Remnant trees and living fences were characteristic of all sites. Each site contained two mist netting arrays, and contiguous blocks of habitat for at least 150 m on each side minimized possible edge effects. Because of their proximity to one another, all sites shared a similar landscape matrix including mature forest, shade coffee, mixed agriculture, pasture and village, so there were no differences in connectivity among these habitats for volant birds. All studies sites were within ∼200 m of a road and ∼50 m of intact forest (see Appendix S1 for an area map).

The study sites forming the chronosequence (with age since disturbance at initiation of study) were La Cueva (two years), La Caoba (five years), Morelia (ten years), and El Corral (20 years); see Appendix S2 for details of this chronosequence. A fifth site in mature dry forest studied as part of a prior project (Latta & Faaborg, 2002) during the winters of 1996–97 through 2000–01 consisted of two transects 2 km apart which were pooled in analyses. This site represented the native habitat of the Mencia sites prior to their deforestation and served as the endpoint in the chronosequence. This control site, Aceitillar (18.098°, −71.635°), was located ∼14 km east of Mencia and at a similar elevation (∼350 m). The Aceitillar site was also affected by occasional grazing by stray cattle and tree cutting for charcoal, but represents some of the best remaining dry forest in the region. Although logistical constraints prevented us from studying the Mencia and Aceitillar sites simultaneously, our extended, multi-year studies encompassed the range of weather conditions typical of the region, and likely ameliorated differences that may have arisen due to annual effects in shorter-term studies.

### La Cueva (two years) and La Caoba (five years)

Vegetation at these sites is similar and dominated by native grasses, forbs, and woody shrubs in height categories <1.5 m, including *Chrysophyllum oliviforme*, *Guettarda preneloupii*, *Chromolaena odorata*, *Trichilia hirta*, *Eugenia monticola*, and *Ehretia tinifolia*. Trees are scarce but consist primarily of very young *Senna spectabilis* (an exotic species) and scattered remnant *Bursera simaruba*. These sites have the lowest diversity and density of shrubs of all sites, as well as the lowest mean canopy height (3.6 m; 3.9 m) and canopy cover (18%; 22%).

### Morelia (ten years)

Vegetative cover occurs primarily in height categories <4.0 m. The diversity of native shrub species is the highest of all study sites, and includes *Chrysophyllum oliviforme*, *Trichilia hirta*, *Psychotria berteroana*, *Piper aduncum*, and *Casearia aculeata*. Trees are small and dominated by the exotic *Senna spectabilis*, but include scattered native *Bursera simaruba*, *Ocotea coriacea*, and *Zanthoxylum martinicense*. Mean canopy height is 5.2 m and mean canopy cover is 54%.

### El Corral (20 years)

Shrub density and diversity decrease but include *Trichilia hirta*, *Casearia aculeate*, *Eugenia monticola*, *Licaria triandra*, and *Nectandra hihua*, all of which are native. Mean canopy height (6.4 m) and canopy cover (76%) increases with native trees that include *Eugenia monticola, Nectandra hihua, Licaria triandra, Acacia farnesiana*, *Bauhinia divaricata,* and *Trichilia pallida,* as well as the introduced species *Leucaena leucocephala.*

### Aceitillar

The mature dry forest site has a mostly closed canopy (94%) with a mean tree height of 10.6 m, a few emergent trees, and an understory dominated by broadleaf shrubs. Trees include *Capparis ferruginea*, *Zizyphus rignoni*, *Bursera simaruba*, *Cameraria angustifolia*, *Cordia buchii*, and *Plumeria obtuse,* which are all native.

### Sampling birds

Protocols were approved by the Institutional Animal Care and Use Committee of the National Aviary/Pittsburgh Zoo and PPG Aquarium (#NA06-004), and field work was authorized by the Secretaria de Estado de Medio Ambiente y Recursos Naturales. We conducted mist-netting in early- (November), mid- (December–January), and late-winter (late-January early-March). At the Mencia sites we used 16 nets (12 m × 30 mm mesh) in each net array for three days: 3 h the first afternoon, all day the next day and 3 h on the final morning. At the Aceitillar site, we placed nets in two lines of 31 and 39 nets with each array covering 700 m. Weather permitting, netting began ∼15 min after sunrise and ended at 16 h 00. All mist-netted birds were identified to species, sexed and aged (juvenile: HY/SY, or adult: AHY/ASY) using plumage or molt limits (Pyle, 1997; Latta et al., 2006). Birds were weighed with a Pesola spring balance (±0.5 g), and we measured wing chord (±0.5 mm). Most birds were banded with a numbered metal band and three color bands to identify each individual. For birds too small to band (hummingbirds, todies) we clipped the outer primary feather so recaptures within a netting session could be identified. Abundances of birds are summarized as birds captured per 1,000 mist-net hrs (mnh), where one 12-m mist net opened for 1 h = 1 mnh.

Mist nets are subject to several biases (Remsen & Good, 1996). This study minimized some of these problems: most vegetation in these sites is relatively low, except in the mature dry forest, yet all sites contain some remnant resulting in similar height ranges. We limited analyses of net capture frequencies to within-species comparisons and assumed equal capture probabilities within species among sites; finally, our mist-netting schedule (2–3 days every six weeks) minimized net shyness of birds.

We classified birds captured in mist nets into groups based on diet on the basis of principal food items consumed in optimal habitats (Wunderle & Latta, 1996). Groups included insectivores, nectarivores, granivores (including frugivores), carnivores, and omnivores. We grouped birds based on migratory status (Latta et al., 2006), including permanent residents and latitudinal migrants. We also classified birds by primary habitat, assigning all species to a single preferred habitat (secondary forest, deciduous forest, evergreen forest) based on Stotz et al. (1996).

### Demographics and site persistence

Abundance data alone can be a misleading indicator of survival and habitat quality (James, 1971). Moreover, among over-wintering migratory birds, dominance interactions result in many species segregating by sex and age class (Holmes, Sherry & Reitsma, 1989; Latta & Faaborg, 2002), so age- and sex-specific data on persistence of individual birds is needed to assess habitat quality (Faaborg et al., 2010). Site persistence has been widely used as a proxy for survival in studies of over-wintering migrants (Holmes, Sherry & Reitsma, 1989; Latta & Faaborg, 2002; Faaborg et al., 2010). Over-winter site persistence is defined as the proportion of birds detected (by resighting or recapture) at any time >24 h after initial capture. Following banding, each plot was systematically searched 56.5 ± 6.2 h (SE) for color-banded individuals, with search areas extending ∼100 m beyond plot boundaries. Although resighting ability may vary with habitat, we minimized this potential bias with the use of multiple expert field personnel with many years of experience resighting color-banded birds in these habitats, and by continuing resighting within a site until all previously identified site-faithful birds were relocated, or until no more newly resighted banded birds were identified after 10 person-hours of searching a site. While a few color-banded individuals may have remained unidentified, consistent resighting effort among sites and years insured comparability of results among habitats.

### Food resources

Assessment of potential food items was focused on insect and spider abundance (hereafter referred to as insects) as we sought to explain the distribution, in particular, of over-wintering migratory birds. Insects were sampled in December, January and February of years 1 and 2 with five yellow sticky traps placed at breast height for 48 h along a transect in each habitat. All insects 0.1–5.0 mm length were counted and pooled across sampling periods; the few larger insects were excluded because most wintering migrants consume primarily small arthropods (Greenberg, 1995; Poulin & Lefebvre, 1996). Leaf litter samples were collected in mid-winter (January) from *n* = 30, 25-cm diameter circles in each site and placed into a sorting pan. All insects <5.0 mm were counted and identified to order.

### Statistical analyses

#### General modeling approach

Because succession from abandoned pasture to mature forests takes decades in tropical dry forests (Murphy & Lugo, 1986) we used a chronosequence approach. While there are inherent limitations to exchanging space for time in this way, chronosequences are essential and robust tools for making inferences about successional processes (DeWalt, Maliakal & Denslow, 2003; Myster & Malahy, 2008; Walker et al., 2010). These limitations are ameliorated somewhat because we are using a direct space-for-time substitution (*sensu* Fukami & Wardle, 2005) with known site histories and each site was sampled repeatedly over five years (Fukami & Wardle, 2005). In particular we can examine change over time both between and within sites (Yanai et al., 2000).

As in other studies of chronosequences we fit models that accommodated the nested nature of multiple measurements from a single site (e.g., Olano, Menges & Martínez, 2006). In particular we fit all models as generalized linear mixed models (GLMMs) with random intercepts whenever appropriate for site (four or five levels), year (five or 11 levels), bird identity (ID) and species (15–25). The random effects used in a model depended on whether community or population-level variables were being analyzed. The number of levels for site and year vary depending on whether only pasture sites were modeled or whether the reference mature forest site is also included. These random effects are partially crossed because all pasture sites were measured in all years, and most species occurred in most sites (Baayen, Davidson & Bates, 2008; West, Welch & Galecki, 2014). For count data (species richness, abundance) we used a Poisson GLMM with an observation-level random effect to account for overdispersion (Harrison, 2014). We used the log of net hours as an offset whenever appropriate to account for variable sampling effort (e.g., models of net captures).

Models were fit in *R* 3.4.4 (R Core Team, 2017) using the *lme4* package (Bates et al., 2015). Because of the relatively low number of levels to our random effects and the nested structure of the data we had to address several model convergence issues. First, as is standard for GLMMs, we centered our predictor variable “pasture age” around zero (Gelman & Hill, 2007). Second, we used the *lme4* extension *blme* (Chung et al., 2013). This package facilitates model convergence by using a modified likelihood function (penalized likelihood) that is mathematically equivalent to using a weakly informative prior for the variance components in a Bayesian model. In particular, *blme* prevents variance components, such as variation between years, from erroneously being estimated as zero. Though this approach borrows from Bayesian approaches, inference is carried out using standard frequentist *p*-values and confidence intervals (Chung et al., 2013; Chung et al., 2015). To further avoid optimization problems we used the *all_fit()* function in the *afex* package (Singmann et al., 2018) to find a numeric optimizer that did not result in convergence warnings from the *lme4* package. The optimizers that produced satisfactory results were usually *Nelder_Mead* and occasionally *nmkb* (Nelder–Mead using derivative-free optimization from the *dfoptim* package (Varadhan, Borchers & ABB Corporate Research, 2017)). Once an appropriate optimizer had been found we checked all convergence criteria as recommended in the *lme4* “*convergence”* helpfile.

It is sometimes recommended to simplify mixed models if random terms are not significant and to improve convergence (Matuschek et al., 2017; but see Barr et al., 2013). We had *a priori* expectations for there to be correlations within sites across years, and within years across sites, and we therefore did not feel it was appropriate to simplify our models and so used the quasi-Bayesian approach advocated by Chung et al. (2013).

To understand how bird communities and traits changed as regeneration time increased we generally fit two sets of models. First, we did regression-style modeling of continuous change over time as regeneration time increased from two to 24 years by using a GLMM to model difference among the La Cueva (two to six years), La Caoba (five to nine years), Morelia (10–14 years), and El Corral (20–24 years) sites. This first set of models did not include the mature forest reference site (Aceitillar) because to our knowledge this site has not been significantly disturbed in historical time. We used this regression-style approach to model both community-level data (e.g., species richness) and species-specific trends. For species-level models we included species as a random effect with a random slope. This allowed us to model both mean change averaged across all species as well as extract estimates of species-specific intercepts and slopes (“best linear unbiased predication”, aka BLUPs; Robins, 1989) from the random effects. We estimated standard errors (SEs) for BLUPs using the *se.coef*() function in the *arm* package (Gelman & Su, 2016) and approximated 95% confidence intervals as ±1.96*SE.

This approach provides several advantages relative to building separate models for each species, including accounting for correlations across species due to common study years and sites shared across species, as well as joint modeling of shared characteristics of species (e.g., migration behavior). These models can both improve power and estimation of trends for rarer species by sharing information across species (“borrowing strength”; Gelman & Hill, 2007; Harrison et al., 2018). It can also reduce Type I errors by alleviating the need for corrections for multiple comparisons (Gelman, Hill & Yajima, 2012). This approach is becoming increasingly popular for multi-species studies (Jackson et al., 2012; Latta et al., 2017).

In addition to our regression-style GLMMs we fit one-way ANOVA-style models which treated time since disturbance (age) as a categorical variable with five levels (four pastures and one forest). If a species was absent from one site we fit the model with just four levels, usually three pastures and the forest site. These models allowed us to make comparisons among the four pasture sites undergoing succession and the mature forest reference site. The two youngest sites had shared values for five and six years post abandonment (Appendix S2) so we removed the overlapping years for these sites. These models were fit both to community-level data (e.g., species richness) and individual species data (e.g., abundance). Species-specific models were fit to only a subset of the more abundant and consistently captured species for which model convergence could be achieved.

To assess change across this chronosequence we did an omnibus likelihood ratio test (LRT) to determine if there was evidence for any difference among sites and then did a focused test for linear (five-level contrast: −2, −1, 0, 1, 2; four-level contrast: 3, −1, 1, 3) and quadratic (five-level contrast: 2, −1, −2, −1, 2; four-level contrast: 1, −1, −1, 1) trends across the chronosequence using a trend contrast (Rosenthal & Rosnow, 1985; Gurevitch & Chester Jr, 1986). Trend tests were conducted using the *multcomp* package (Hothorn, Bretz & Westfall, 2008).

Depending on how a model was parameterized we usually assessed the significance of focal parameters using likelihood ratio tests (LRTs) and report *χ*^2^ and *p*-values as *P*_LRT_, though occasionally we use Wald *t*-statistics (*P*_*t*_). We confirmed significant *p*-values using parametric bootstrapping via the *lme4* function *bootMermod*(). For plotting, we calculated 95% confidence intervals (95% CI) around overall GLMM regression lines using the *predictInterval()* function in the *merTools* package (Knowles & Frederick, 2016). Means for each site were estimated using the GLMM and confidence intervals approximated using standard errors (SE) from the model coefficients (95% CI = ±1.96*SE). We built all plots using *ggplot2* (Wickham, 2009) and *cowplot* (Wilke, 2017).

### Comparing communities

We used rarefaction to compare species richness among sites, pooling data from across years. Rarefaction produces idealized species-accumulation curves that allow direct comparison of results among groups that differ in patterns of abundance or are sampled using different techniques (Gotelli & Colwell, 2001). Rarefaction calculates the expected species richness of the different groups for a constant sampling effort, but does not provide an estimate of asymptotic richness. Rather, for each accumulation curve we calculated a Chao 1 non-parametric estimator of richness with its variance and 95% confidence interval (Chao, 1984).

We calculated species richness and Shannon Diversity for each site and each year of the study. We converted diversity to the “effective number of species” by exponentiating species richness (Jost, 2006) to represent true diversity with mathematical properties allowing comparison among groups. We also calculated numerical dominance of species captured within each site, and Pielou’s evenness index (Magurran, 1988; Pielou, 1966) of the distribution of individuals among taxa (absolute evenness = 1.0). We modeled how these indices varied as pastures aged using regression and how they varied across the chronosquence using one-way ANOVA. We log-transformed species diversity and logit transformed evenness because it is a percentage (Warton & Hui, 2011) and modeled both using a linear mixed model (LMM). Species richness was modeled using a Poisson GLMM with an observation-level random affect to account for overdispersion (Harrison, 2014). All test statistics are reported for transformed data and analyses were re-run on untransformed data for plotting.

For each site we pooled data from across years and used Jaccard’s index to compare the similarity of communities based on presence/absence of species, and Sorenson’s measure to compare the similarity of sites based on the proportional abundance of recorded species. Both indices were calculated using the *SimilarityPair()* function in the *SpadeR* package (Chao et al., 2016). We also used non-metric multidimensional scaling (NMDS) with a Bray-Curtis distance matrix in the *vegan* package (Oksanen et al., 2017) to visualize the overlap in community composition among the sites. Due to the repeated sampling of the same sites and variation in effort these results are only exploratory.

### Species-specific comparisons among habitats

Species-specific trends in capture rates were estimated using a random-slopes Poisson GLMM. To examine non-linear trends indicated by these models we also built separate GLMMs for certain species that incorporated a quadratic time term. We also built separate one-way ANOVA models for each species to examine differences between the pasture sites and the mature reference forest.

We modeled the site persistence of birds captured during the first year of the study at pasture sites (2003–2004) and mature forest (1996–1997). A bird was considered site persistent if it was re-sighted or recaptured during the year it was first captured or any subsequent year. We modeled site persistence using a one-way ANOVA style binomial GLMM where the number of “trials” was the number of individuals of each species captured, and the number of “successes” was the number of site-persistent birds. We calculated mean site persistence of migrants and residents for each site using species and month of capture (November, January, or February/March) as random effects. We included an observation-level random effect to account for binomial overdispersion (Harrison, 2015). We tested for linear and quadratic trends for both migrants and residents.

We examined differences among sites in the proportion of individual birds that were adult, male, migratory, and endemic. We analyzed all of these variables as binary data with logistic regression GLMMs. As for capture rates, we examined species-specific trends as regeneration time increased using a single random-slopes GLMM, and variation between the pasture and reference forest using one-way ANOVA. The species used in a given model depended on how accurately they could be aged or sexed and sample size. For models of the proportions of migrants and endemics only bird ID (for recaptures) and year were used as random effects.

To determine how the abundance of flying and litter arthropods changed as pastures underwent succession we modeled total arthropod abundance versus the initial time since pasture abandonment using a nested Poisson GLMM, with site as a random effect and an observation-level random effect for overdispersion. We tested for an overall difference between pasture sites and the mature forest using a *t*-test like Poisson GLMM with an indicator variable for “pasture” and “non-pasture”, with site treated as a random effect; confidence intervals around this difference were calculated using parametric bootstrapping. We explored overall differences in the arthropod assemblage by treating each sample as an independent replicate in a multivariate analysis of variance (MANOVA) with site as a factor. We examined trends in individual taxonomic groups across the chronosequence by plotting mean percent compositions of each taxa with bootstrapped 95% confidence intervals.

To determine if body condition varied among sites we calculated the Scaled Mass Index (SMI or ${\hat {M}}_{I}$; Peig & Green, 2009). We used log-transformed body mass and wing chord measurements as our body size variables and the *smatr* package (Warton et al., 2012) to calculate the scaling exponent *b*_*SMA*_. SMI was only calculated for individuals that were classified as site persistent. In contrast to our analysis of site persistence, we used site-persistent birds captured any time during the study for this analysis. If a bird was captured more than once, we only used mass and length data from its first capture. We first built a regression-style linear mixed model (LMM) to estimate species-specific changes in SMI as regeneration time increased. We then examined change across the chronosequence using one-way ANOVA. Because of limited sample sizes we used only linear models without random effects for these ANOVA analyses.

## Results

### Patterns of bird distribution

Through 40,723 mnh we recorded 7,315 captures of 60 species (Table 1). Twenty-five species of birds with >30 captures each composed 96.9% of all captures, but capture rates were higher in the two-year and five-year-old sites (231.5 and 236.0 birds/1,000 mnh, respectively) than in the ten-year and twenty-year-old sites (152.5 and 132.9 birds/1,000 mnh, respectively), or the mature dry forest (155.4 birds/1,000 mnh).

**Table 1:** Occurrence of birds in four habitats of differently aged regenerating dry forest and mature dry forest in the Sierra de Bahoruco, Dominican Republic.

**Species**	**Code**	**Status**^a^	**Pref. Habitat**^b^	**Diet**^c^	**La Cueva (2 years)**		**La Caoba (5 years)**		**Morelia (10 years)**		**El Corral (20 years)**		**Aceitillar (mature)**	
					**Sum**	**Caps /1,000 mnh**	**Sum**	**Caps /1,000 mnh**	**Sum**	**Caps /1,000 mnh**	**Sum**	**Caps /1,000 mnh**	**Sum**	**Caps /1,000 mnh**
Sharp-shinned Hawk (*Accipiter striatus*)	SSHA	R	EF	C	1	0.13					2	0.29	2	0.18
American Kestrel (*Falco sparverius*)	MAKE	R	SF	C	1	0.13			1	0.13				
Northern Potoo (*Nyctibius jamaicensis*)	NOPO	R	SF	I	1	0.13								
Zenaida Dove (*Zenaida aurita*)	ZEDO	R	DF	G									5	0.44
Mourning Dove (*Zenaida macroura*)	MODO	R	SF	G	2	0.26								
Common Ground-Dove (*Columbina passerina*)	CGDO	R	SF	G	230	30.22	167	22.90	101	13.37	98	14.11	28	2.47
Key West Quail-Dove (*Geotrygon chrysia*)	KWQD	RE	DF	G	1	0.13					14	2.02	19	1.68
Ruddy Quail-Dove (*Geotrygon montana*)	RUQD	R	EF	G	1	0.13	2	0.27	2	0.26			4	0.35
White-fronted Quail-Dove (*Geotrygon leucometopia*)	WFQD	R	EF	G	2	0.26			2	0.26				
Yellow-billed Cuckoo (*Coccyzus americanus*)	YBCU	R	DF	I										
Mangrove Cuckoo (*Coccyzus minor*)	MACU	R	DF	I	6	0.79	10	1.37	8	1.06	3	0.43		
Hispaniolan Lizard-Cuckoo (*Coccyzus longirostris*)	HILC	RE	DF	C	21	2.76	17	2.33	17	2.25	30	4.32	26	2.30
Smooth-billed Ani (*Crotophaga ani*)	SBAN	R	SF	O	2	0.26	5	0.69	1	0.13				
Burrowing Owl (*Athene cunicularia*)	BUOW	R	SF	C					2	0.26				
Antillean Mango (*Anthracothorax dominicus*)	ANMA	R	EF	N	45	5.91	26	3.57	48	6.36	26	3.74	44	3.89
Hispaniolan Emerald (*Chlorostilbon swainsonii*)	HIEM	RE	EF	N	26	3.42	14	1.92	16	2.12	3	0.43	6	0.53
Vervain Hummingbird (*Mellisuga minima*)	VEHU	R	SF	N	5	0.66	8	1.10	10	1.32	1	0.14	5	0.44
Hispaniolan Trogon (*Priotelus roseigaster*)	HITR	RE	EF	O					1	0.13				
Broad-billed Tody (*Todus subulatus*)	BBTO	RE	DF	I	73	9.59	88	12.07	93	12.31	102	14.68	92	8.13
Narrow-billed Tody (*Todus angustirostris*)	NBTO	RE	EF	I	1	0.13	7	0.96					4	0.35
Antillean Piculet (*Nesoctites micromegas*)	ANPI	R	DF	I			3	0.41	2	0.26	1	0.14	10	0.88
Hispaniolan Woodpecker (*Melanerpes striatus*)	HIWO	RE	EF	I	1	0.13							1	0.09
Hispaniolan Pewee (*Contopus hispaniolensis*)	HIPE	RE	EF	I	1	0.13				1	0.14		17	1.50
Stolid Flycatcher (*Myiarchus stolidus*)	STOF	R	DF	O	28	3.68	9	1.23	23	3.05	18	2.59	43	3.80
Gray Kingbird (*Tyrannus dominicensis*)	GRAK	R	SF	I	12	1.58	2	0.27	2	0.26	1	0.14		
Black-whiskered Vireo (*Vireo altiloquus*)	BWVI	R	DF	I	17	2.23	2	0.27	5	0.66			13	1.15
Rufous-throated Solitaire (*Myadestes genibarbis*)	RTSO	R	EF	F	4	0.53	4	0.55	1	0.13				
Wood Thrush (*Hylocichla mustelina*)	WOTH	M	EF	O									1	0.09
Red-legged Thrush (*Turdus plumbeus*)	RLTH	R	DF	O	62	8.15	59	8.09	31	4.10	19	2.74	85	7.51
Gray Catbird (*Dumetella carolinensis*)	GRCA	M	SF	O			2	0.27						
Northern Mockingbird (*Mimus polyglottos*)	NOMO	R	SF	O	39	5.12	40	5.48	21	2.78	20	2.88	5	0.44
Palmchat (*Dulus dominicus*)	PALM	RE	SF	F	3	0.39	3	0.41	4	0.53				
Ovenbird (*Seiurus aurocapilla*)	OVEN	M	EF	I	105	13.79	112	15.36	185	24.49	144	20.73	134	11.84
Louisiana Waterthrush (*Parkesia motacilla*)	LOWA	M	EF	I			1	0.14						
Blue-winged Warbler (*Vermivora pinus*)	BWWA	M	SF	I	1	0.13								
Black-and-white Warbler (*Mniotilta varia*)	BAWW	M	EF	I	9	1.18	7	0.96	17	2.25	36	5.18	73	6.45
Tennessee Warbler (*Oreothlypis peregrina*)	TEWA	M	SF	I					1	0.13				
Common Yellowthroat (*Geothlypis trichas*)	COYE	M	SF	I	29	3.81	149	20.43	31	4.10			1	0.09
Hooded Warbler (*Setophaga citrina*)	HOWA	M	EF	I			1	0.14					1	0.09
American Redstart (*Setophaga ruticilla*)	AMRE	M	EF	I	72	9.46	65	8.91	32	4.24	22	3.17	37	3.27
Cape May Warbler (*Setophaga tigrina*)	CMWA	M	EF	N	112	14.71	185	25.37	45	5.96	15	2.16	88	7.77
Northern Parula (*Setophaga americana*)	NOPA	M	EF	I	3	0.39	2	0.27	3	0.40			2	0.18
Magnolia Warbler (*Setophaga magnolia*)	MAWA	M	EF	I	7	0.92	1	0.14	4	0.53	4	0.58	2	0.18
Blackpoll Warbler (*Setophaga striata*)	BLPW	M	SF	I			1	0.14						
Black-throated Blue Warbler (*Setophaga caerulescens*)	BTBW	M	EF	I	69	9.07	64	8.78	88	11.65	75	10.80	45	3.98
Palm Warbler (*Setophaga palmarum*)	PAWA	M	SF	I	24	3.15	13	1.78	4	0.53	3	0.43	1	0.09
Yellow-rumped Warbler (*Setophaga coronata*)	YRWA	M	EF	I			1	0.14						
Prairie Warbler (*Setophaga discolor*)	PRAW	M	SF	I	31	4.07	40	5.48	11	1.46	1	0.14	3	0.27
Green-tailed Ground-Tanager (*Microligea palustris*)	GTGT	RE	EF	I	37	4.86	68	9.32	23	3.05	47	6.77	124	10.96
Bananaquit (*Coereba flaveola*)	BANA	R	EF	N	136	17.87	96	13.16	58	7.68	23	3.31	291	25.71
Black-crowned Palm-Tanager (*Phaenicophilus palmarum*)	BCPT	RE	SF	O	88	11.56	62	8.50	72	9.53	83	11.95	141	12.46
Summer Tanager (*Piranga rubra*)	SUTA	M	SF	O			1	0.14						
Hispaniolan Spindalis (*Spindalis dominicensis*)	HISP	RE	EF	F	16	2.10	15	2.06	4	0.53			
Yellow-faced Grassquit (*Tiarus olivaceus*)	YFGR	R	SF	G	266	34.95	176	24.13	70	9.27	59	8.49	7	0.62
Black-faced Grassquit (*Tiarus bicolor*)	BFGR	R	SF	G	28	3.68	44	6.03	13	1.72	10	1.44	9	0.80
Greater Antillean Bullfinch (*Loxigilla violacea*)	GABU	R	EF	O	142	18.66	146	20.02	98	12.97	62	8.93	387	34.19
Rose-breasted Grosbeak (*Pheucticus ludovicianus*)	RBGR	M	SF	G									3	0.27
Blue Grosbeak (*Guiraca caerulea*)	BLGR	M	SF	G					1	0.13				
Indigo Bunting (*Passerina cyanea*)	INBU	M	SF	G	1	0.13	2	0.27						
Nutmeg Mannikin (*Lonchura punctulata*)	NUMA	R	SF	G	1	0.13								

**Notes.**

a R, Resident; RE, Resident endemic; M, Neotropical Migrant.

b EF, Evergreen forest; DF, Deciduous Forest; SF, Secondary forest, pature and edge. From Latta et al. (2006).

c C, Carnivore; F, Frugivore; G, Generalist; I, Insectivore; N, Nectarivore; O, Omnivore. From  Latta, Rimmer & McFarland (2003).

**Figure 1:**
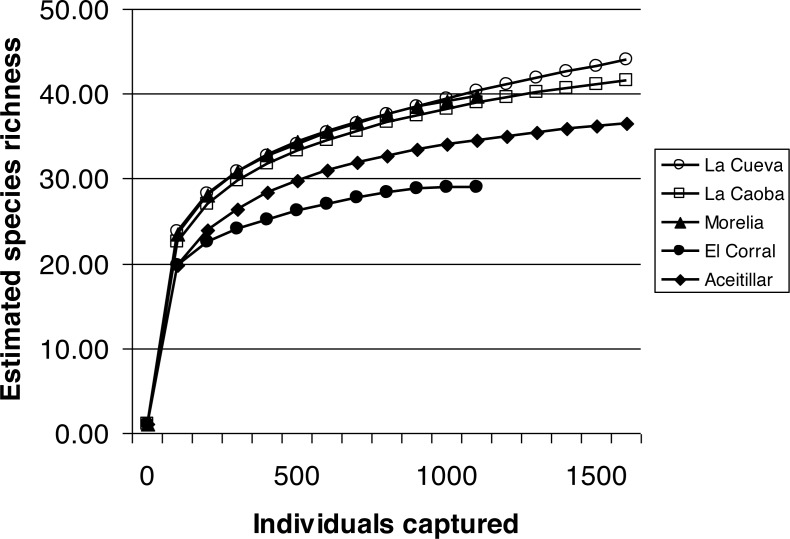
Estimated species richness in a chronosequence of dry forest regenerating from pasture with two years of regeneration at La Cueva, five years at La Caoba, ten years at Morelia, twenty years at El Corral, and mature dry forest at Aceitillar. Analyses of Chao 1 indicators of diversity indicate no significant difference in species pools among the sites.

Rarefaction curves indicated that sampling intensity was appropriate, with curves with pooled data from all years for each site approaching an asymptote (Fig. 1). Observed species richness was highest in the three youngest sites and lowest in the twenty-year-old habitat, with intermediate richness in the mature dry forest (Fig. 1; Table 2). Shannon Diversity Index was highest in the three early-successional sites; diversity was lowest in the mature dry forest (Table 2). Chao 1 non-parametric estimates of species richness indicate no significant difference in the size of the species pool among sites, although the Chao 1 point estimate of richness was highest in the youngest site (Table 2).

**Figure 2:**
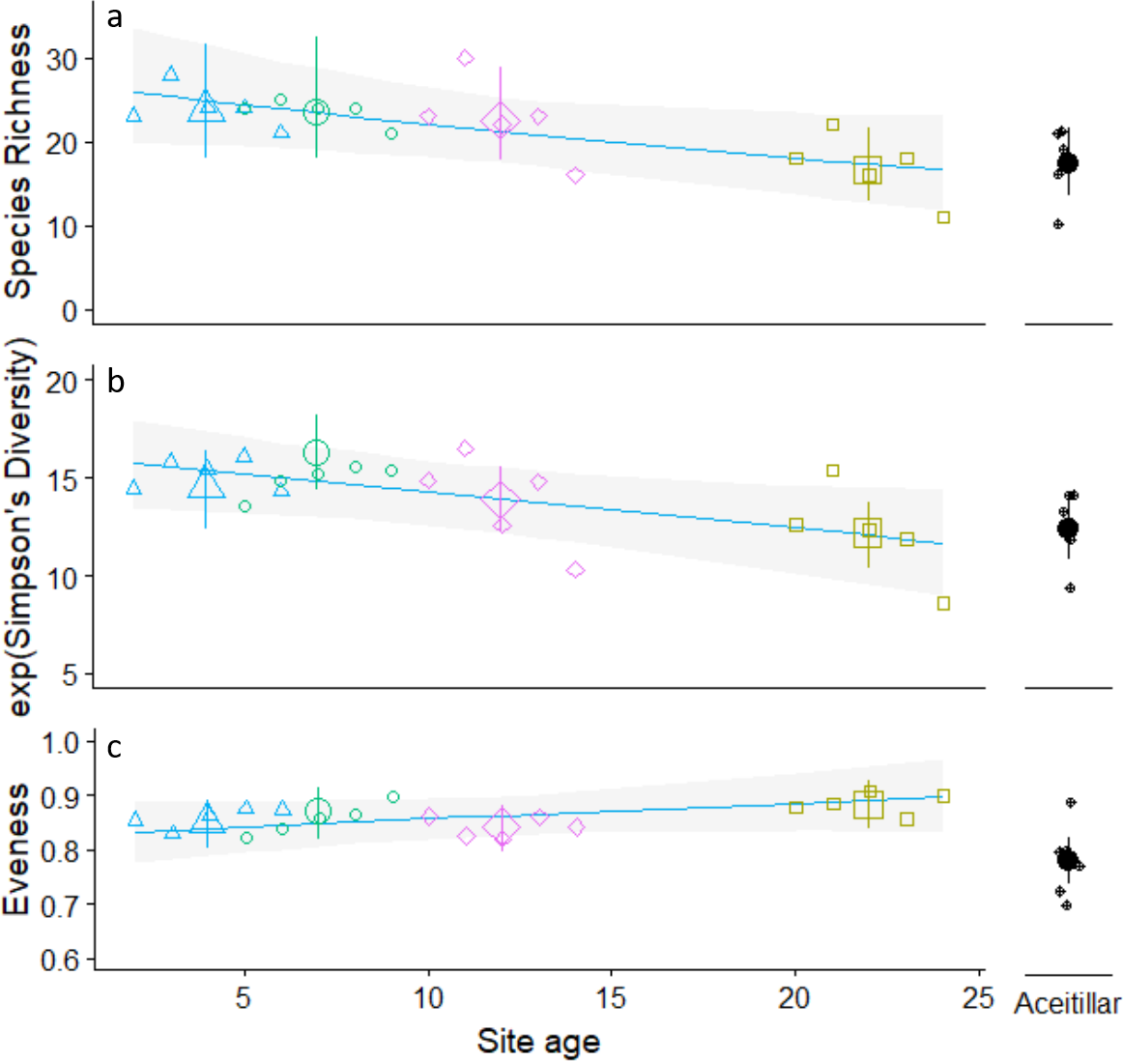
Trends in observed annual species richness (A) Simpson’s diversity (B) Pielou’s evenness (C). Across pastures of different ages regenerating to dry forest. Regression lines calculated using only data from regenerating pastures and site means calculated using a one way ANOVA type model that included the mature forest site (Aceitillar). Site means for the two youngest sites were calculated using ages that did not overlap (La Cueva: two to four years of regeneration; La Caoba seven to nine years of regeneration). Error bars are estimated 95% confidence intervals.

Regression analysis indicated that species richness (*β*_site_age_ =  − 0.02, ${\chi }_{4,5}^{2}=5.7$, *P*_LRT_ = 0.017) and diversity (*β*_site_age_ =  − 0.18, ${\chi }_{4,5}^{2}=9.6$, *P*_LRT_ = 0.072) decreased significantly as habitat regeneration time increased (Fig. 2; Appendix S3). While the mature forest site was generally similar to the oldest regenerating pasture, ANOVA trend analysis indicated that these declines were consistent across the entire chronosequence (species richness trend: *Z* =  − 2.4, *P* = 0.02; diversity trend: *Z* =  − 5.6, *p* < 0.001). In contrast, evenness increased (*β*_site_age_ = 0.24, ${\chi }_{4,5}^{2}=4.2$, *P*_LRT_ = 0.04) with regeneration time, but declined to its lowest value at the mature forest site (quadratic trend: *Z* =  − 2.4, *P* = 0.017).

**Table 2:** Mist-net capture rates, species richness, and diversity of birds in regenerating dry forest and mature dry forest in the Sierra de Bahoruco, Dominican Republic.

	La Cueva	La Caoba	Morelia	El Corral	Aceitillar
	2 years	5 years	ten years	20 years	Mature
Elevation	395 m	410 m	400 m	400 m	340 m
Mist net hours	7,612	7,293	7,553	6,946	11,319
Capture rate^a^	231.5	235.8	152.4	132.9	155.4
Migrant capture rate^a^	60.8	88.7	55.9	43.2	34.5
Resident capture rate^a^	170.7	147.1	96.5	89.7	120.8
Species richness (indivs)	45 (1,762)	42 (1,720)	40 (1,151)	29 (923)	37 (1,759)
Shannon diversity index	2.97	2.97	2.96	2.77	2.66
Effective number of species	19.5	19.5	19.3	16.0	14.3
Chao 1 Estimator (SD)	65.2 (12.6)	45 (2.7)	43.6 (3.2)	41.5 (11.6)	41.2 (4.0)
Chao 1 confidence interval	40.4–90.0	39.6–50.4	37.2–50.0	18.8–64.2	33.3–49.0
Evenness	0.78	0.79	0.80	0.82	0.74
Resident spp (indivs)	33 (1,299)	25 (1,073)	28 (729)	21 (623)	24 (1,368)
% resident individuals	73.7%	62.4%	63.3%	67.5%	77.8%
Endemic spp (indivs)	11 (269)	9 (277)	10 (234)	7 (267)	9 (421)
% endemic individuals	15.3%	16.1%	20.3%	28.9%	23.9%
Migrant spp (indivs)	12 (463)	17 (647)	12 (422)	8 (300)	13 (391)
% migrant individuals	26.3%	37.6%	36.7%	32.5%	22.2%

**Notes.**

a Birds captured/1,000 mnh.

For data pooled across years, the extent of numerical dominance of species within a site also suggests the degree of evenness (Table 3); the five dominant species in each site accounted for 47.9–52.9% of captures in all early-successional habitats, whereas five species accounted for 61.1% of captures in mature dry forest. Although Greater Antillean Bullfinch (scientific names of all birds appear in Table 1) appeared as one of the five most abundant species in all habitats, other species contributed to among-habitat differences in numerical dominance. For example, Yellow-faced Grassquit and Common Ground-Dove were among the most common species in the two-year and five-year-old sites, with Ovenbird most abundant in the ten-year and twenty-year-old sites, and Bananaquit, Black-crowned Palm-Tanager, and Green-tailed Ground-Tanager reaching highest densities in oldest sites.

**Table 3:** Dominance rank (proportion of all captures) of five most abundant species in regenerating dry forest and mature dry forest. Scientific names of species are found in Table 1.

Species	La Cueva	La Caoba	Morelia	El Corral	Aceitillar
	2 years	5 years	10 years	20 years	Mature
Common Ground-Dove	2 (13.0)	3 (9.7)	2 (8.8)	3 (10.6)	
Broad-billed Tody			4 (8.1)	2 (11.0)	
Ovenbird			1 (16.1)	1 (15.6)	4 (7.6)
Common Yellowthroat		4 (8.7)			
Cape May Warbler	5 (6.4)	1 (10.8)			
Black-throated Blue Warbler			5 (7.6)		
Green-tailed Ground-Tanager					5 (7.0)
Bananaquit	4 (7.7)				2 (16.5)
Black-crowned Palm-Tanager				4 (9.0)	3 (8.0)
Yellow-faced Grassquit	1 (15.1)	2 (10.2)			
Greater Antillean Bullfinch	3 (8.1)	5 (8.5)	3 (8.5)	5 (6.7)	1 (22.0)

**Figure 3:**
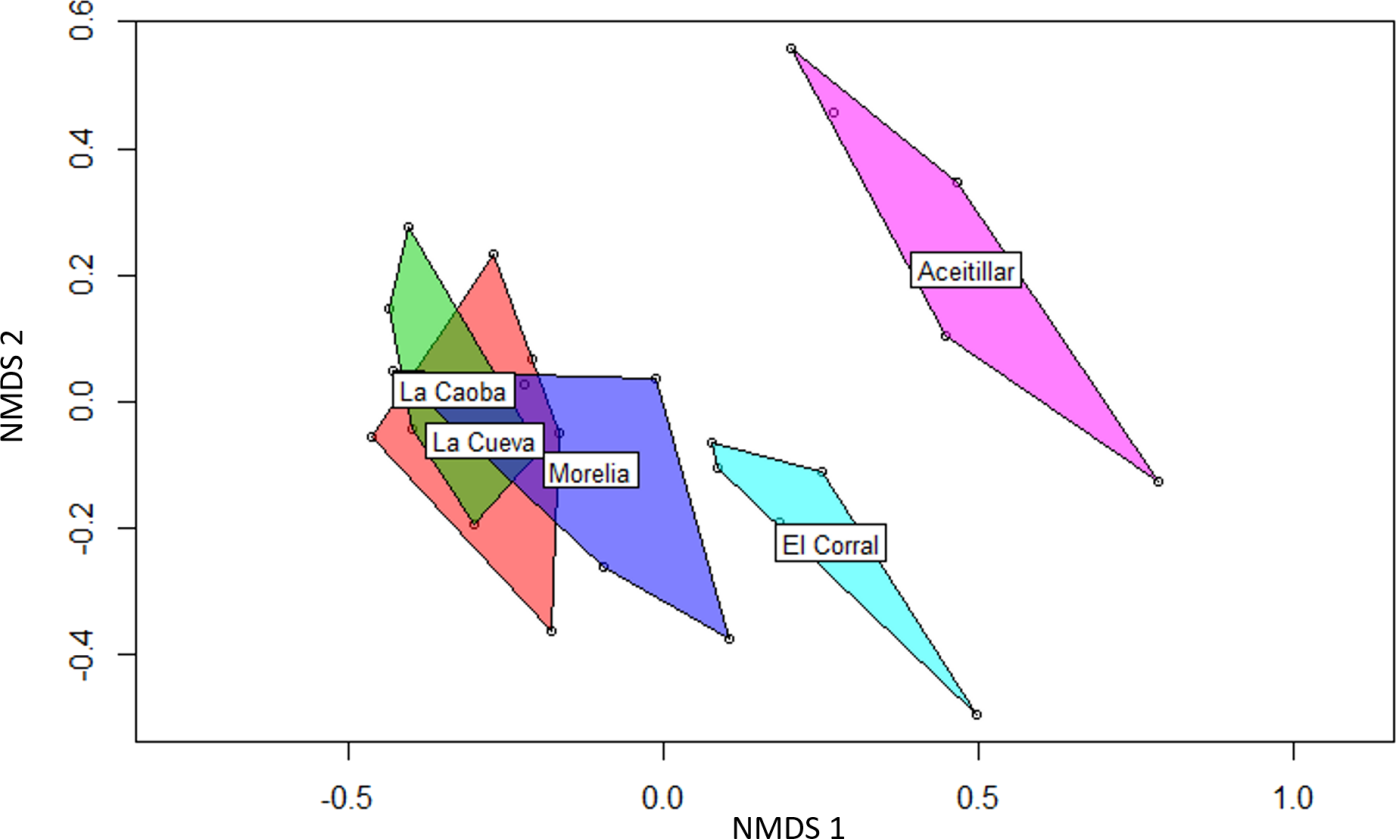
Non-metric multidimensional scaling (NMDS) biplot showing differences in bird species composition in four regenerating pastures and one mature native dry forest. Non-metric multidimensional scaling (NMDS) biplot using Bray-Curtis distances showing differences in bird species composition in four regenerating pastures and one mature native dry forest in southern Hispanola. The regenerating pastures began the five year study at the following ages: La Caoba, two years; La Cueva, five years; Morelia, 10 years; El Corral, 20 years.

Similarity indices based on species presence/absence pooled across years were only moderately high with scores of 0.50–0.69 for habitat pairs (Table 4); scores tended to be higher between similarly-aged sites, especially among the three youngest pastures. Sorenson similarity based on the proportional abundances of individuals mist-netted (Table 4) had a broader range (0.67–0.81). Exploratory ordination with NMDS also indicated that the youngest sites had highly overlapping species pools, and that the mature dry forest reference site was very distinct; the oldest abandoned pasture was also somewhat distinct from the younger sites (Fig. 3).

**Table 4:** Similarity indices of birds captured in five sites based on species presence/absence (Jaccard Similarity; unshaded portion), and proportional abundances of individuals mist-netted (Sorenson Index; shaded portion). Data were pooled across years. Values in parentheses are bootstrapped 95% confidence intervals.

	**La Cueva**	**La Caoba**	**Morelia**	**El Corral**	**Aceitillar**
**La Cueva**		0.78 (0.71–0.86)	0.81 (0.74–0.88)	0.74 (0.66–0.82)	0.71 (0.62–0.79)
**La Caoba**	0.64 (0.55–0.74)		0.81 (0.74–0.88)	0.70 (0.63–0.77)	0.7 (0.62–0.77)
**Morelia**	0.68 (0.59–0.78)	0.67 (0.58–0.77)		0.7 (0.63–0.77)	0.67 (0.6–0.74)
**El Corral**	0.59 (0.5–0.68)	0.54 (0.45–0.62)	0.54 (0.46–0.62)		0.81 (0.73–0.9)
**Aceitillar**	0.55 (0.45–0.65)	0.53 (0.45–0.62)	0.5 (0.42–0.58)	0.69 (0.58–0.8)	

Of the 60 species recorded, 13 (22%) were endemic residents, and 22 (37%) were latitudinal migrants (Tables 1 and 2). The proportion of endemic individuals increased as succession in the pastures progressed, but this trend was not significant (*β*_site_age_ = 0.06, *SE* = 0.04, *Z* = 1.4, *P*_LRT_ = 0.15), yet across the entire chronosequence there was a significant linear trend in the frequency of endemic individuals (*Z* = 5.28, *P* < 0.001). Though their community composition was different as discussed above, the frequency of endemic individuals was similar in the oldest pasture (25%) and the mature forest (23%).

The proportion of migrant individuals varied from 26–46% in the pasture sites (Tables 1 and 2). The overall proportion of migrants increased as regeneration time increased (*β*_site_age_ = 0.01, *SE* = 0.03) but the trend was not significant in regression models (${\chi }_{4,5}^{2}=0.25$, *P*_LRT_ = 0.61). Capture rates of migrants declined from the second oldest to the oldest site but there was no significant quadratic trend (${\chi }_{5,6}^{2}=0$, *P*_LRT_ = 1). The proportion of migrants in the mature forest was lower than any other site (15%), resulting in a significant negative quadratic trend across the chronosequence (*Z* =  − 5.25, *P* < 0.001).

Presence/absence data pooled across years suggest that most species occurring at our study sites were habitat generalists. Of the 60 recorded species, 21 of 25 species with *n* > 30 captures were recorded in all five sites (Table 1). Hispaniolan Lizard-Cuckoo and Black-crowned Palm-Tanager appeared to be true habitat generalists with no overall differences in capture rates among sites. However, others showed differences in abundance among sites (Table 1). Among residents, six species appeared to prefer the early-successional sites. These included Common Ground-Dove, Hispaniolan Emerald, Northern Mockingbird, Hispaniolan Spindalis, and Yellow-faced and Black-faced grassquits. In contrast, three species showed higher capture rates in more mature, forested sites. These included Key West Quail-Dove, Green-tailed Ground-Tanager, and Greater Antillean Bullfinch.

### Linear trends as pastures age

**Figure 4:**
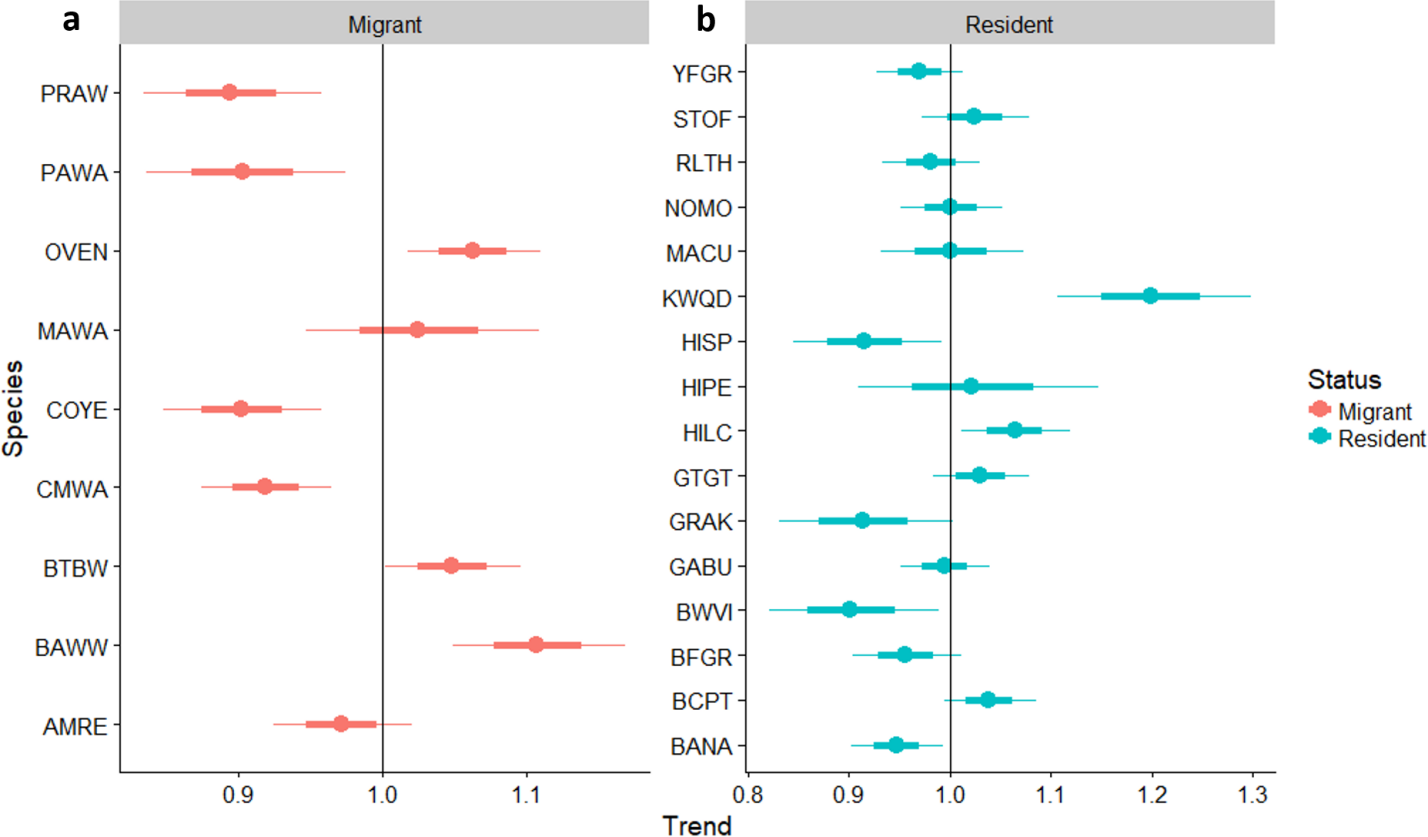
Trends in abundance for individual migrant and resident species as pastures regenerate to dry forest. Trends in abundance for: (A) migratory species, (B) permanent resident species, as pastures regenerate to dry forest. A trend of 1.0 indicates no net change. Thick error bars are standard errors (SE) and thin error bars are approximate 95% confidence intervals (1.96*SE). Species codes can be found in Table 1.

Neotropical migrants varied in their response to forest regeneration. Capture rates of Ovenbird, Black-throated Blue Warbler, and Black-and-white Warbler all increased significantly as regeneration time increased (Fig. 4). Prairie Warbler, Palm Warbler, Common Yellowthroat, and Cape May Warbler all showed evidence of significant overall declines in capture rates as sites regenerated, while American Redstart showed a marginal decline. Among resident species, the Key West Quail-Dove and Hispaniolan Lizard-Cuckoo had higher capture rates as regeneration time increased, while captures of the Green-tailed Ground-Tanager and Black-crowned Palm-Tanager increased marginally. Capture rates of Hispaniolan Spindalis, Gray Kingbird, Black-whiskered Vireo, and Bananquit declined significantly with regeneration time.

### Trends including mature forest reference

Across the entire chronosequence, the Black-and-white Warbler was the only Neotropical migrant which increased consistently in abundance with habitat regeneration time (ANOVA trend test: *P*_linear_ = 0.009, *P*_quadratic_ = 0.98; Appendices S4 and S5). Most other species increased in abundance over part of the chronosequence but declined in the oldest pasture and/or the mature forest site as indicated by significant quadratic trends (American Redstart: *P*_linear_ < 0.001, *P*_quadratic_ = 0.083; Black-throated Blue Warbler *P*_linear_ < 0.001, *P*_quadratic_ < 0.001; Ovenbird *P*_quadratic_ < 0.001). The Cape May Warbler (*P*_linear_ = 0.001), Prairie Warbler (*P*_linear_ < 0.001), and Palm Warbler (*P*_linear_ = 0.0013) exhibited consistent declines across the entire sequence of habits. Most Neotropical migrants reached peak abundance at the La Caoba site which represented five years of regeneration when the study began (Appendices S4 and S5).

Because many of the migratory species had non-linear trends in abundance across sites, we fit additional regression models to data for individual species that included a quadratic term age of regeneration. Ovenbird (*P* = 0.01), Common Yellowthroat (*P* = 0.06), Black-throated Blue Warbler (*P* = 0.06), and Palm Warbler (*P* = 0.01) all had significant quadratic terms in these regression models, indicating peaked abundances at a single pasture site with subsequent declines in abundance (Appendix S6).

### Age ratios

**Figure 5:**
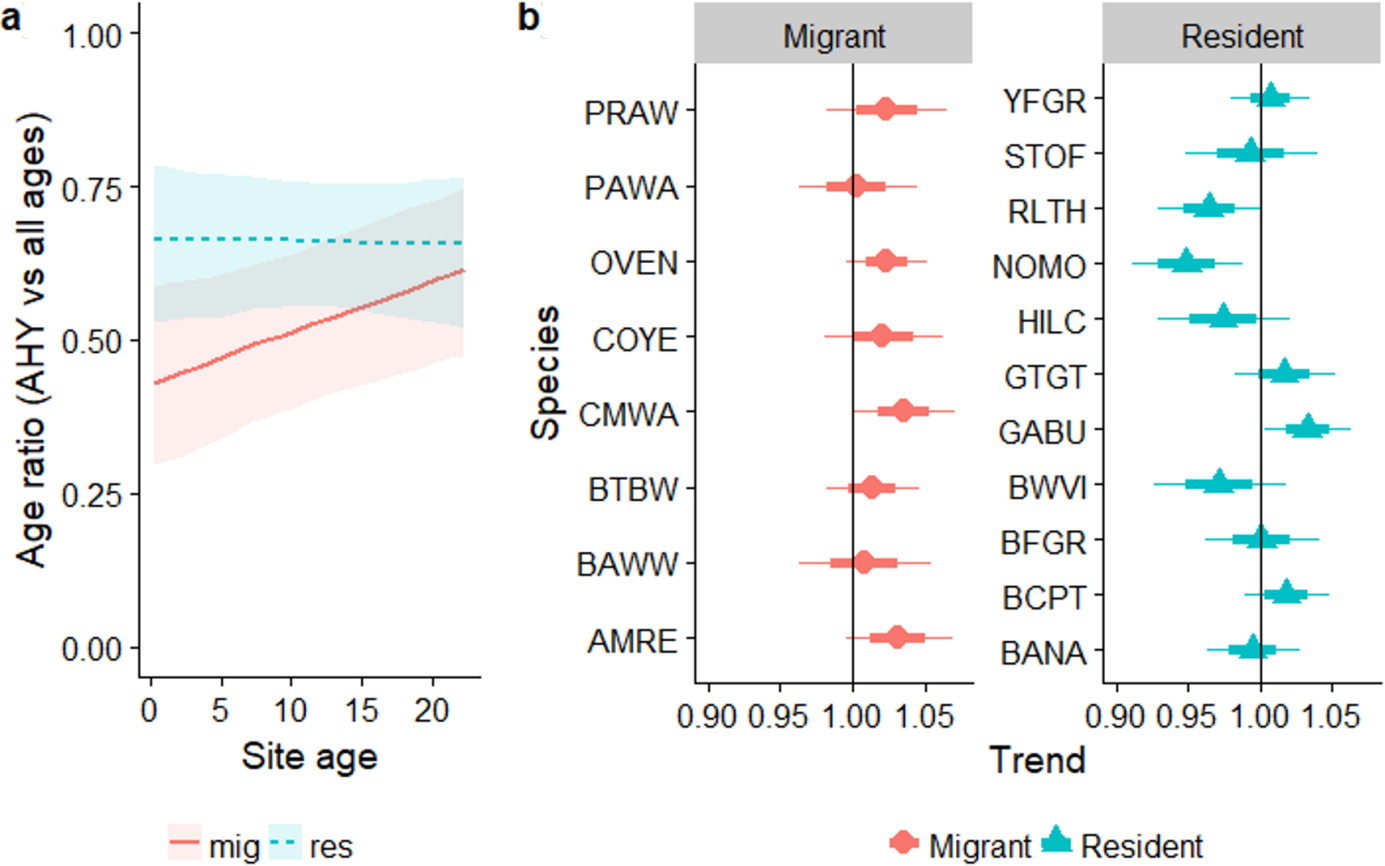
Trends in the proportion of birds aged AHY (after hatch year) or older occurring in a chronosequence of dry forest regenerating from pasture. Data are: (A) averaged across all species; (B) for individual species. In (B), a trend of 1.0 indicates no net change. Thick error bars are standard errors (SE) and thin error bars/error bands are approximate 95% confidence intervals (1.96*SE). Species codes can be found in Table 1.

Overall, the age ratio of permanent resident species remained constant as forest regenerated (*β*_site_age_ =  − 0.002, *SE* = 0.014, *Z* =  − 0.17, *P*_Wald_ = 0.9), while for over-wintering migrants the proportion of older individuals increased as regeneration progressed (Fig. 5A; residency × site-age interaction: *β*_site_age_ = 0.035, *SE* = 0.02, *χ*_9,10_ = 4.48, *P*_LRT_ = 0.03). The age ratios of individual resident species increased or decreased with regeneration time to various degrees (Fig. 5B), while the trend for all migrant species was for a larger proportion of older birds as regeneration progressed. Increases in the proportion of older birds with regeneration time were significant for Cape May Warbler and marginally significant for Ovenbird and American Redstart (Fig. 5B).

Similar to the regression analysis, across the entire chronosequence there was no evidence for any trend for age ratios of residents (linear trend: *P* = 0.23; quadratic trend: *P* = 0.87). For migrants, there was a significant quadratic trend (*p* = 0.006) driven by very low mean proportions of AHY migrants in the reference mature forest at Aceitillar (33%) relative to the abandoned pasture sites (43%–61%). Mean proportions of AHY birds at each site for the most abundant species are summarized in Appendix S7.

### Sex ratios

**Figure 6:**
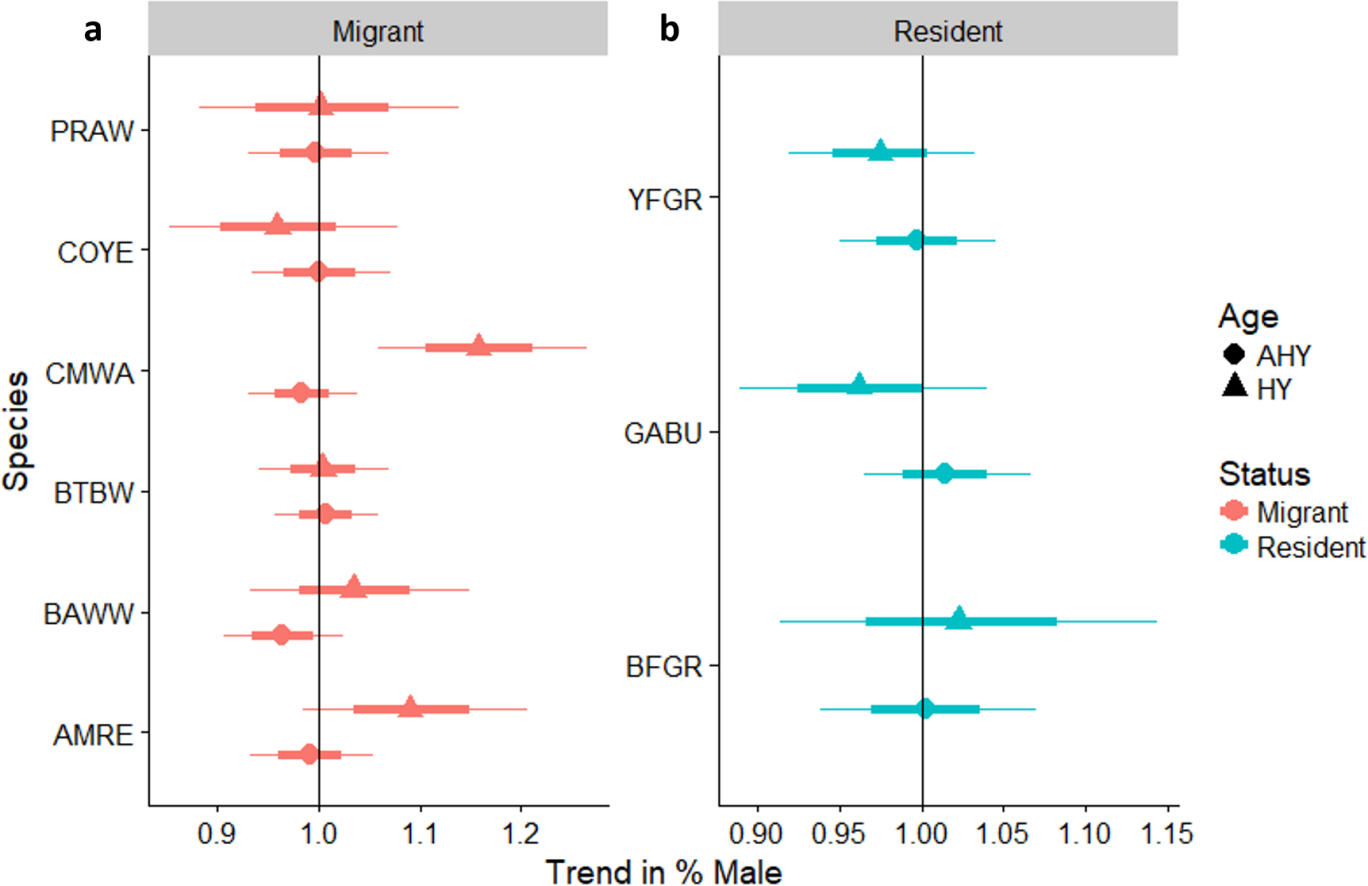
Trends in the percentage of male birds by age class occurring in a chronosequence of dry forest regenerating from pasture; trends in the percentage of male (A) migrants, and (B) residents, by age class, occurring in a chronosequence of dry forest regenerating from pasture. HY, Hatch Year; AHY, After Hatch Year. A trend of 1.0 indicates no net change. Thick error bars are standard errors (SE) and thin error bars/error bands are approximate 95% confidence intervals (1.96*SE). Species codes can be found in Table 1.

We reliably determined sex of six species of over-wintering migrants that were mist-netted with adequate sample sizes for analysis, including Common Yellowthroat, American Redstart, and Cape May, Black-throated Blue, Black-and-white, and Prairie warblers (Fig. 6A). We also determined sex ratios of three permanent resident species: Greater Antillean Bullfinch, Yellow-faced Grassquit, and Black-faced Grassquit (Fig. 6B). For most individual migrant species there was no change in the sex ratio as regeneration time increased. Two exceptions were significant increases in the proportion of male Cape May Warblers, and a marginal increase in American Redstarts. Overall, the sex ratio of residents tended to be more male-biased than migrants (58% male vs. 36% male; *β*_residency_ = 0.92, *SE* = 0.40, *χ*_7,8_ = 4.6, *P*_LRT_ = 0.03). Considering the entire chronosequence, the percentage of males captured did increase for migrants (linear trend *Z* = 2.3, *P* = 0.022; Appendix S3), but not for residents (*Z* =  − 0.5, *P* = 0.6). Mean sex ratios birds for the most abundant species are summarized for each site in Appendix S7.

### Preferred diet

**Figure 7:**
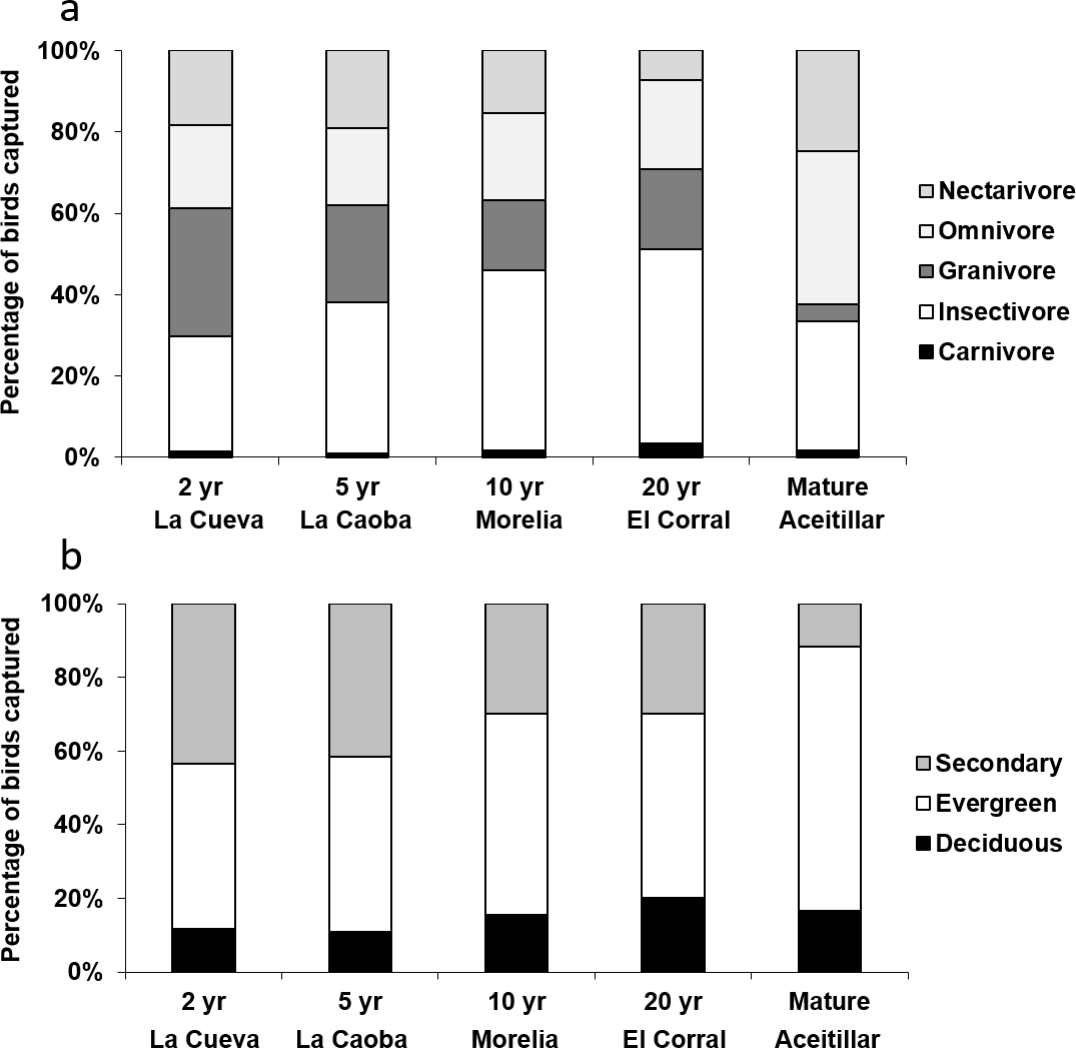
Proportions of individuals captured in mist nets categorized by: (A) diet, and (B) preferred habitat type. Captures took place in dry forest regenerating after agricultural clearance with plots representing two years of regeneration at La Cueva (1,762 birds), five years at La Caoba (1,720 birds), ten years at Morelia (1,151 birds), twenty years at El Corral (923 birds), and mature dry forest (1,759 birds).

Exploratory multivariate analysis with NMDS indicated that a major axis of variation among sites was the number of omnivorous individuals. The proportion of individuals of omnivorous species increased over the first 15 years of succession (*β*_site∗age_ = 0.016, *SE* = 0.0068, *χ*_4,5_ = 4.34, *P*_LRT_ = 0.037). Across the entire chronosequence, the smallest proportion of insectivorous individuals occurred in the most recently abandoned pasture (23%, bootstrapped 95% CI [17%–32%]; Fig. 7A) and the largest occurred in the mature forest (43%, 95% CI [35%–52%]). This resulted in an overall significant linear increase across the chronosequence (*Z* = 3.6, *P* = 0.004).

### Preferred habitats

The proportion of individuals of species which preferred secondary forests declined over the first 15 years of succession (*β*_site_age_ =  − 0.07, *SE* = 0.03, *χ*_4,5_ = 3.73, *P*_LRT_ = 0.054), but increased somewhat at the oldest regenerating site (quadratic site age effect: *β*_site_age^∧^2_ = 0.005, *SE* = 0.002, *χ*_5,6_ = 10.4, *P*_LRT_ = 0.001). Across the entire chronosequence, the highest proportion of secondary-forest birds occurred in the most recently abandoned pasture (38%; Fig. 7B), and the least occurred in the mature forest (13%). This resulted in an overall significant linear trend across the chronosequence (*Z* =  − 6.6, *P* < 0.001). At the mature forest site, most individuals were of species that preferred evergreen forests (75%).

### Site persistence

Within the regenerating pastures, mean site persistence of all migrant species increased from 0.44 at the youngest site to 0.62 at the oldest, but dropped to 0.15 at the mature forest site. This resulted in an overall significant negative quadratic trend (*Z* =  − 4.2, *P* < 0.001). For resident species, site persistence declined significantly overall (*Z* =  − 2.6, *P* = 0.009) from the youngest site (0.38) to the mature forest (0.13), though persistence was highest in the oldest regenerating site (0.43). Mean site persistence rates for the most abundant species are summarized in Appendix S8.

### Arthropod resources

**Figure 8:**
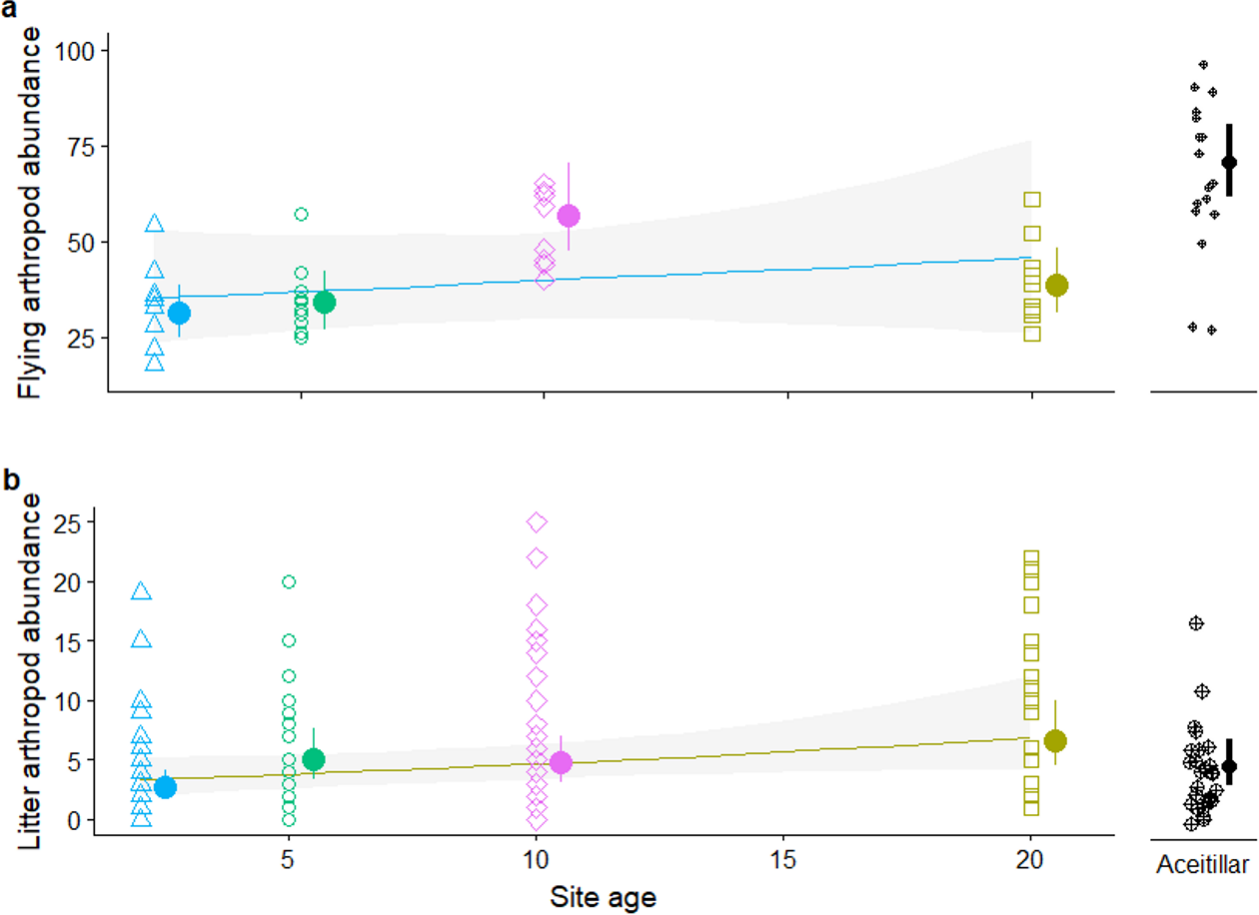
Abundance of insects captured on: (A) sticky traps. (B) leaf litter traps. Error bars and band are approximate 95% CI. The *Y* axis in plot (B) was truncated at 25, removing three outliers.

Site persistence may be related to food availability; flying insects caught on sticky traps were least abundant in the two-year-old habitat and most abundant in the mature forest site (Fig. 8A), with flying insects 1.7 times (bootstrapped 95% CI [1.02–2.6]) as abundant in the mature forest as compared to the pastures (nested-GLMM: ${\chi }_{3,4}^{2}=4.67$, *P*_LRT_ = 0.03). Across just the pasture sites, flying insects increased consistently with age of the regenerating site (Fig. 8A), although the trend was not significant (*β* = 0.013, *SE* = 0.017, ${\chi }_{3,4}^{2}=0.62$, *P*_LRT_ = 0.43). Exploratory MANOVA analyses treating samples within sites as independent indicates variation in the composition of insect communities among sites (*F*_32,164_ = 1.98, *P* = 0.003). The two most recently abandoned pastures (<10 years since abandonment) tended to have more Coleoptera and Homoptera, and fewer Diptera, than the two older sites (>10 years) and the mature forest (Appendix S9a).

Leaf litter arthropods were most abundant in the oldest abandoned pasture, and least abundant in the mature forest (Fig. 8B). Leaf litter insects were 0.55 times (bootstrapped 95% CI [0.22–1.32]) as abundant in the mature forest compared to the pastures, although the difference was only marginally significant (${\chi }_{3,4}^{2}=2.69$, *P*_LRT_ = 0.10). Across pasture sites, litter arthropod abundance increased marginally with age (*β* = 0.039, *SE* = 0.022, ${\chi }_{3,4}^{2}=4.03$, *P*_LRT_ = 0.045). Exploratory analyses indicate marginally significant variation in the composition of insect communities among sites (*F*_28,168_ = 1.48, *P* = 0.0.07). Ants tended to increase with time since abandonment, but had relatively low abundance in the forest, while spiders tended to decline with time but were relatively abundant in the forest (Appendix S9b).

**Figure 9:**
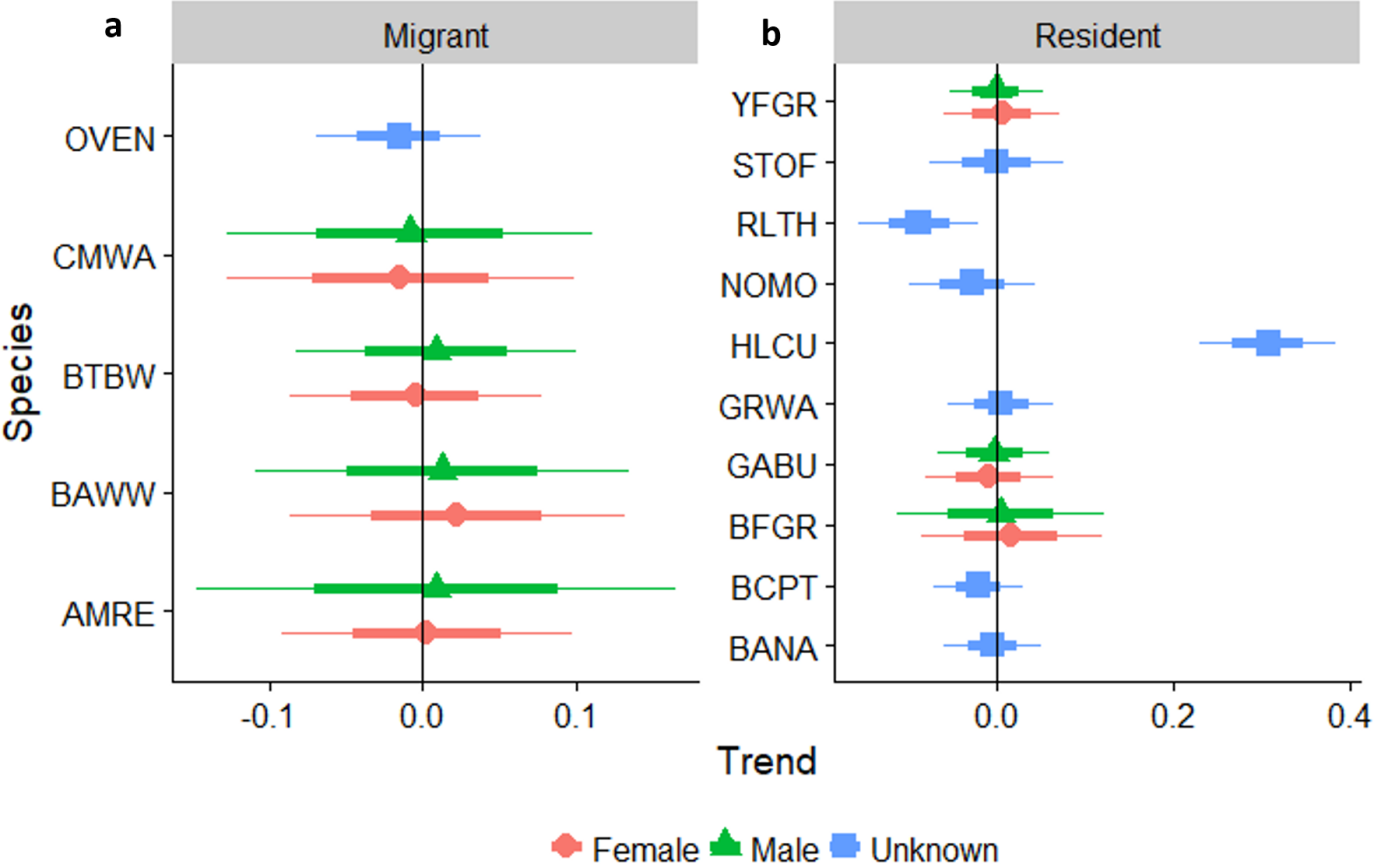
Trends in body condition for individual (A) migratory, and (B) resident species, a occurring in a chronosequence of dry forest regenerating from pasture. A trend of 0 indicates no net change. Thick error bars are standard errors (SE) and thin error bars are approximate 95% confidence intervals (1.96*SE). Species codes can be found in Table 1.

### Body condition

Body condition of site-persistent individuals of 15 species was compared across pasture sites by analyzing variation in the scaled mass index (SMA). As sites regenerated, regression analysis indicated that condition increased significantly for Hispaniolan Lizard-Cuckoo, and decreased for Red-legged Thrush (Fig. 9). For species that occurred in both abandoned pastures and the mature forest site, ANOVA trend analysis indicated that Bananquit peaked in body condition at moderately regenerated sites but declined in condition in the oldest regenerating habitat and in the mature forest (linear trend: *t* =  − 2.65, *P* = 0.009; quadratic trend: *t* =  − 2.51, *P* = 0.013; Appendices S10 and S11). Black-crowned Palm-Tanager did not show a significant decline in regression analysis (Fig. 9), but did when considering the mature forest site (linear trend: *t* =  − 3.4, *P* = 0.001). Similarly, the abundance of the Greater Antillean Bullfinch was fairly constant as regeneration time increased (Fig. 9), but declined significantly at the mature forest site (linear trend: *t* =  − 3.4, *P* = 0.001; quadratic trend: *t* =  − 2.3, *P* = 0.023). Green-tailed Ground-Tanager showed a similar pattern, with a relatively constant condition as regeneration progressed, but condition declined for birds at the mature forest site (linear trend: *t* =  − 2.32, *P* = 0.021; quadratic trend: *t* =  − 1.7, *P* = 0.09). Among over-wintering migratory birds, adjusted body mass did not vary significantly across sites (Fig. 9, Appendices S8 and S9).

## Discussion

Despite the similarity in species richness and diversity across these sites, we found that early successional and mature dry forests provide habitat for unique sets of permanent resident and over-wintering migratory birds. By looking beyond simple presence/absence, most species showed a preference for either relatively younger or generally older habitats. These differences were likely driven by differences in resource availability and foraging preferences, as trophic composition differed between young and old sites, reflecting differences in availability of resources or foraging sites (Blake & Loiselle, 1991; Blake & Loiselle, 2001).

Many of the species occurring in the early-successional sites are considered typical of open areas and young, shrubby second-growth, and are granivores or frugivores. These species include Common Ground-Dove, and Yellow-faced and Black-faced grassquits. In addition, the few island nectarivores frequently had higher capture rates in early successional sites; the Antillean Mango, Hispaniolan Emerald, Cape May Warbler, and Bananaquit took advantage in particular of remnant *Bursera* trees, prolifically flowering *Senna* trees, and other understory vegetation (D Mejía, pers. obs., 2003–2008). These results are consistent with other studies that show bird assemblages in tropical agroecosystems are composed of disproportionately more frugivorous and nectarivorous birds and fewer insectivorous species than native forest (Tscharntke et al., 2008). Results showing significantly higher adjusted body mass in early-successional habitats for the Bananaquit and the omnivorous Black-crowned Palm-Tanager reflect similar patterns in site persistence for these species, and help explain why tropical agroecosystems, including regenerating pastures, are composed of disproportionately more omnivorous and nectarivorous birds, and fewer insectivores compared to native forest (Karr et al., 1990; Blake & Loiselle, 2001; Tscharntke et al., 2008).

In addition to supporting species typically associated with early-successional habitats, shrubs have also been shown to provide seasonal opportunities for foraging on fruit and critical cover for birds thought of as primary forest species (Anders, Faaborg & Thompson, 1998; Streby et al., 2011; Stoleson, 2013). For example, the occasional presence in early-successional habitats of species such as White-fronted Quail-Dove, Ruddy Quail-Dove, and Rufous-throated Solitaire, typical of primary forest, suggests that individuals may move temporarily from nearby older forests in the landscape matrix, and into early-successional habitats such as La Cueva and La Caoba where shrub density is high, to take advantage of unique food resources (Blake & Loiselle, 1991).

The twenty-year-old and mature forest sites were, in contrast, distinguished by insectivores, such as Ovenbird and Green-tailed Ground-Tanager, as well as more omnivorous species, such as Greater Antillean Bullfinch, as seen in other tropical forested habitats (Blake & Loiselle, 2001). High insect abundance in mature dry forest, in particular, may explain the relatively high capture rates of such species as Hispaniolan Pewee, Black-and-white Warbler, and Green-tailed Ground-Tanager, but fail to explain lower adjusted body mass in this same habitat. The ground-tanager, a species which forages low in the understory (Latta et al., 2006), may respond negatively to depressed numbers of ground-dwelling insects since our studies revealed lower counts of arthropods in leaf litter samples in mature dry forest. In addition, many of these ground-dwelling insects, such as ants, are considered less palatable for birds (Zach & Falls, 1979; Bell, 1990).

An especially important finding from our study is that Hispaniolan endemics comprise a greater proportion of mist net captures in the oldest habitats, and especially in mature dry forest. While some endemics, such as Hispaniolan Lizard-Cuckoo and Black-crowned Palm-Tanager, appear to be true generalists across the studied habitats (Latta et al., 2006), older sites were characterized by robust populations of several more unique species, such as Key West Quail-Dove, Green-tailed Ground-Tanager, and Greater Antillean Bullfinch. The uniqueness of these older habitats is apparent in results from the Sorenson Index which emphasizes the relatively low similarity of the twenty-year-old and mature forest habitat to other sites. Our findings are similar to those of MacGregor-Fors & Schondube (2011) who also found that simplified agricultural landscapes such as cattle pastures had bird communities with endemic species poorly represented compared to tropical dry forests in Mexico.

Over-wintering migrants are an especially significant portion of the avian community in the five-year and ten-year-old sites with a dense understory. These include species known to prefer shrubby and open habitats, such as Common Yellowthroat (Lynch, 1992), Palm Warbler (Latta, 2003) and Prairie Warbler (Latta & Faaborg, 2001), but also the frequently nectarivorous Cape May Warbler (Latta & Faaborg, 2002), which took advantage of abundant flowering trees and shrubs, including *Senna* and *Bursera*. Only the ground-foraging Ovenbird, which prefers shaded sites with abundant leaf litter, and Black-and-white Warbler, which forages for arthropods on trunks and branches of larger trees (Wunderle & Latta, 1996), favored the older regenerating and the mature forest sites. Our results support the conclusion that second-growth and other disturbed habitats can be important to many species of long-distance migrants (Lynch, 1992; Wunderle & Waide, 1993).

However, relatively young, regenerating dry forest appears to be suboptimal-quality habitat for most latitudinal migrants based on age and sex ratios and site persistence. A preponderance of behaviorally dominant male and AHY migrants has been used as an indicator of high-quality habitat for many species (Faaborg et al., 2010), and segregation has been demonstrated previously for American Redstart, Cape May Warbler, Black-throated Blue Warbler, and Prairie Warbler (Faaborg et al., 2010). Among migrants in this study we found populations tended to be skewed towards females and HY/SY individuals, suggesting that these early successional habitats may be of relatively low quality for species such as Common Yellowthroat, Palm Warbler, and Prairie Warbler, and this deserves additional study. However, because the ratio of older birds increased with habitat age for Cape-May Warbler, Ovenbird, and American Redstart, older habitats in our chronosequence may have been approaching the optimum for these species.

That our younger regenerating sites represented suboptimal habitat for most latitudinal migrants is supported by low rates of site persistence for migrants when compared with comparable data from previous studies. For example, site persistence of warblers wintering in native forests and shade coffee plantations range from 52 to 80% for American Redstart (Holmes, Sherry & Reitsma, 1989; Sherry & Holmes, 1996; Wunderle & Latta, 2000), 42–85% for Black-throated Blue Warbler (Holmes, Sherry & Reitsma, 1989; Wunderle & Latta, 2000), 61–82% for Black-and-white Warbler (Wunderle & Latta, 2000), 67–88% for Cape May Warbler (Latta & Faaborg, 2002), and 75–84% for Prairie Warbler (Latta & Faaborg, 2001). In all cases, warblers over-wintering in our early-successional sites exhibited site persistence levels at the low end of these ranges.

### Conservation implications

Many previous studies of the impact of anthropogenic habitat change on birds have focused on the impact of deforestation and fragmentation of primary forests on species and communities and the associated loss of biodiversity from these landscapes (Stouffer & Bierregaard, 1995; Sekercioglu et al., 2002). Relatively few studies, however, have looked at the inverse questions: i.e., what is the pattern of regained bird diversity as agricultural lands are abandoned, when do regenerating forests attain equivalent ecological value for birds found in mature forest, and what components of the agricultural matrix support higher biodiversity?

Unfortunately, our results are not particularly encouraging in terms of immediate impacts from creating new habitat for birds in Hispaniolan dry forest landscapes. That the avian community in our mature dry forest is highly distinctive as compared to forests regenerating for up to 24 years, emphasizes that attaining ecological equivalency takes decades or even longer. Regenerating forests appear to have more value to over-wintering migratory birds, which occurred in large numbers, but age and sex ratios, and site persistence data, also suggest that early-successional habitats may be of relatively low quality for most species. Some optimism may be seen, however, in that sites occurring later in our chronosequence tended to have older migrants present, and thus may provide appreciably better habitat for a few migrant species.

Results from this study suggest some components of the agricultural matrix may be of particular importance in contributing to avian diversity in regenerating dry forest sites. While tree cover in the context of the agroecological matrix may typically be thought of in terms of remaining blocks of primary forest, individual trees may also be retained for fruit, shade, or other functions (Harvey et al., 2006). These remnant trees may then serve birds as dietary resources (Sekercioglu et al., 2007), shelter (MacGregor-Fors & Schondube, 2011), stepping stones between forest patches (Graham, 2001; Sekercioglu et al., 2007), and microclimatic refuges (Greenberg, Bichier & Sterling, 1997; Sekercioglu et al., 2007). Despite the difference in age of regenerating pastures, a variety of bird species were likely attracted to the abundant flowers and fruit of remnant trees remaining in all of the abandoned pastures, contributing to diversity indices. A variety of trees have been shown to be important for birds in an agricultural countryside (Harvey et al., 2006; Estrada, Coates-Estrada & Meritt, 1997; Sekercioglu et al., 2007), but leguminous *Acacia* like *Senna* (Greenberg, Bichier & Sterling, 1997), and *Bursera* (Greenberg, Foster & Marquez-Valdelamar, 1995; Latta, Gamper & Tietz, 2001), both of which occurred in our sites, have been shown to be particularly valuable to birds. This suggests that agricultural efforts and restoration methods that promote the retention of remnant trees in the landscape can have a very positive impact on many bird species.

Finally, although protecting natural vegetation is essential for the preservation of major elements of biodiversity (Bruner et al., 2001; McKinney, 2002; Sekercioglu, Daily & Ehrlich, 2004), our results suggest that the agricultural matrix can provide habitat for some birds in the buffer zone of forested protected areas. Discussions of buffering the effects of agriculture on protected areas have often focused on providing a mature forest matrix to facilitate the movement or dispersal of forest species (Vandermeer & Perfecto, 2007; Burkey, 1989; Vandermeer & Carvajal, 2001). Until now much less has been written on how the agroecological matrix itself, and early successional regeneration, can provide habitat for birds (but see MacGregor-Fors & Schondube, 2011). However, care should be taken to examine body condition and demographic rates as we do here in assessing the value of these early successional habitats.

### Study limitations

For this study we have opportunistically made use of a chronosequence of abandoned pastures that could be surveyed in parallel over five years, and combined this information with a previously studied mature forest site. Making inference from chronosequences necessarily requires assuming that the successional trajectories that resulted in conditions at the surveyed sites will be similar at other places and times. In our study we have additionally assumed that our reference forest site, which has never been logged, represents a comparable endpoint to pasture succession. Numerous factors could result in different successional trajectories in this landscape, such as the availability of propagules of forest species (Holl, 1999), the presence of invasive species (Aide et al., 2000; Sobanski & Marques, 2014), and altered soil properties (Nagy et al., 2017; Lozano-Baez et al., 2018). However, these factors are often most important during the initiation of woody cover regeneration, and our sites had all begun at least the initial development of woody cover. In addition, despite the recognized limitations of chronosequences, they have generally been considered invaluable for understanding long-term ecological dynamics (DeWalt, Maliakal & Denslow, 2003; Myster & Malahy, 2008; Walker et al., 2010).

## Conclusions

In conclusion, this study shows that the regenerating forests that we examined failed to provide habitat comparable to mature dry forest even after 24 years of regeneration, as indicated by distinct suites of bird species in the contrasting habitats in our chronosequence, including the presence of some unique Hispaniolan endemics in mature habitats. Remnant overstory trees and dense understory in early successional regenerating pastures, however, do provide habitat for a suite of species, including many over-wintering migrants. But demographic and site persistence data suggest that these habitats may not be optimal for many migrants in particular. Because some of our regenerating sites were >20 years old and still failed to replicate mature dry forest habitat, regaining complex microhabitats and structures such as canopy closure in these dry forest sites may take decades or longer. Therefore, while early successional habitats may be viewed as part of a complex mosaic of habitats capable of attracting many birds, perhaps their greatest value is as a component of the buffer zone, enhancing biodiversity conservation through integration with protected areas which themselves contain mature forests that harbor more unique, often endemic, bird species.

##  Supplemental Information

10.7717/peerj.5217/supp-1 Appendix S1 Map of study site (a) The Dominican Republic with locations of the four Mencia pasture sites and Aceitillar broadleaf reference site; (b) the Pedernales region; (c) location of four study sites near Mencia, Dominican Republic including La Cueva (abandoned for 2 yr when the study began), La Caoba (5 yr), Morelia (10 yr), and El Corral (20 yr).

10.7717/peerj.5217/supp-2 Appendix S2 Temporal and age relationships for a chronosequence of four abandoned pastures and a mature dry forest reference site near Mencia, Dominican Republic

10.7717/peerj.5217/supp-3 Appendix S3 Results of Generalized Linear Mixed Model (GLMM) analysis of change in bird community and population characters across a chronosequence of four abandoned pastures and a mature dry forest reference site near Mencia, Dominican Republic

10.7717/peerj.5217/supp-4 Appendix S4 Results of test of 1-way ANOVA-style models for linear and quadratic trends in the abundance of individual Neotropical migrant species

10.7717/peerj.5217/supp-5 Appendix S5 Modeled capture rates from 1-way ANOVA style generalized linear mixed models for migrant species at four pasture sites and one reference forest (Aceitillar)

10.7717/peerj.5217/supp-6 Appendix S6 Significance of “pasture age” terms in regression-type generalized linear mixed models (GLMM) examining non-linear patterns in bird abundance

10.7717/peerj.5217/supp-7 Appendix S7 Demographics of overwintering Neotropical migrants, residents, and endemics in regenerating broadleaf habitat of different ages, and in mature dry forest in the Sierra de Bahoruco, Dominican Republic.

10.7717/peerj.5217/supp-8 Appendix S8 Percentage of captured individuals (n) of 10 resident species that were site persistent in regenerating pastures and mature dry forest in the Sierra de Bahoruco, Dominican Republic.

10.7717/peerj.5217/supp-9 Appendix S9 Percentage composition of flying insects captured on sticky traps (top) and terrestrial insects in leaf litter samples (bottom) from four abandoned pasture sites and a mature forest reference

10.7717/peerj.5217/supp-10 Appendix S10 Results of linear and quadratic trend tests of scaled mass index (SMI) from 1-way ANOVA style linear models for migrant and resident species at four pasture sites and one reference forest (Aceitillar)

10.7717/peerj.5217/supp-11 Appendix S11 Modeled mean scaled mass index (SMI) from 1-way ANOVA style linear models for migrant species at four pasture sites and one reference forest (Aceitillar)

## Additional Information and Declarations

### Field Study Permissions

The following information was supplied relating to field study approvals (i.e., approving body and any reference numbers): Permission to work in the Sierra de Bahoruco, Dominican Republic was provided by letters from the Secretaria de Estado de Medio Ambiente y Recursos Naturales.

### Data Availability

The following information was supplied regarding data availability: GitHub: https://github.com/brouwern/DRmencia.
