# Supplementary figures and images for: Avian community characteristics and demographics reveal how conservation value of regenerating tropical dry forest changes with forest age
Source: PeerJ. 2018 Jul 10;6:e5217. doi: 10.7717/peerj.5217 (PMC6044266; doi:10.7717/peerj.5217)

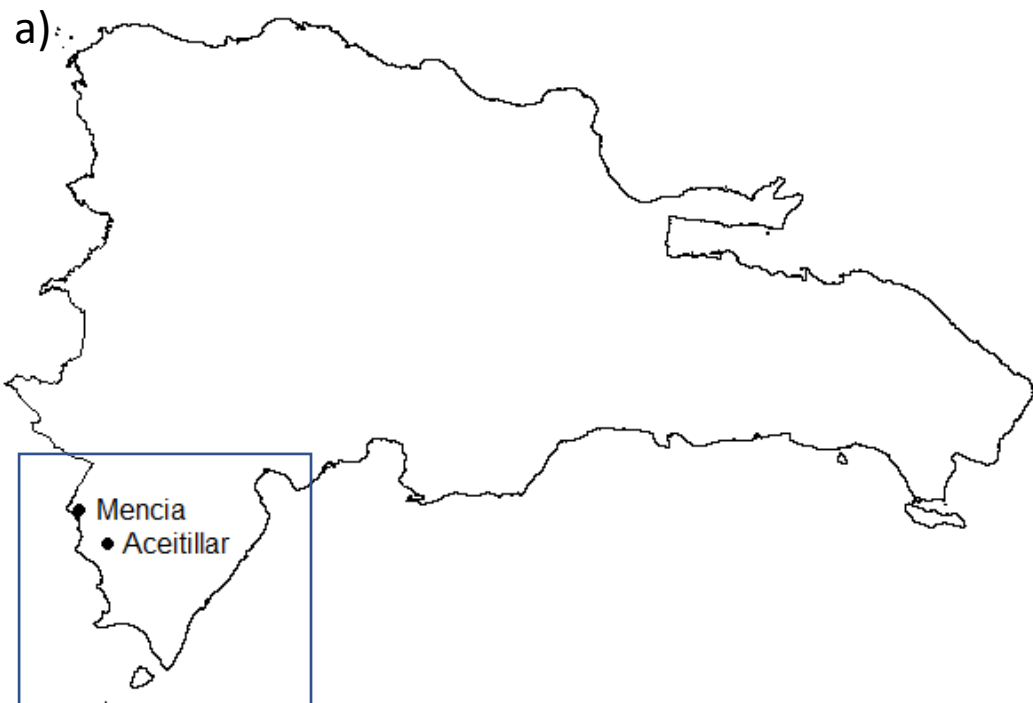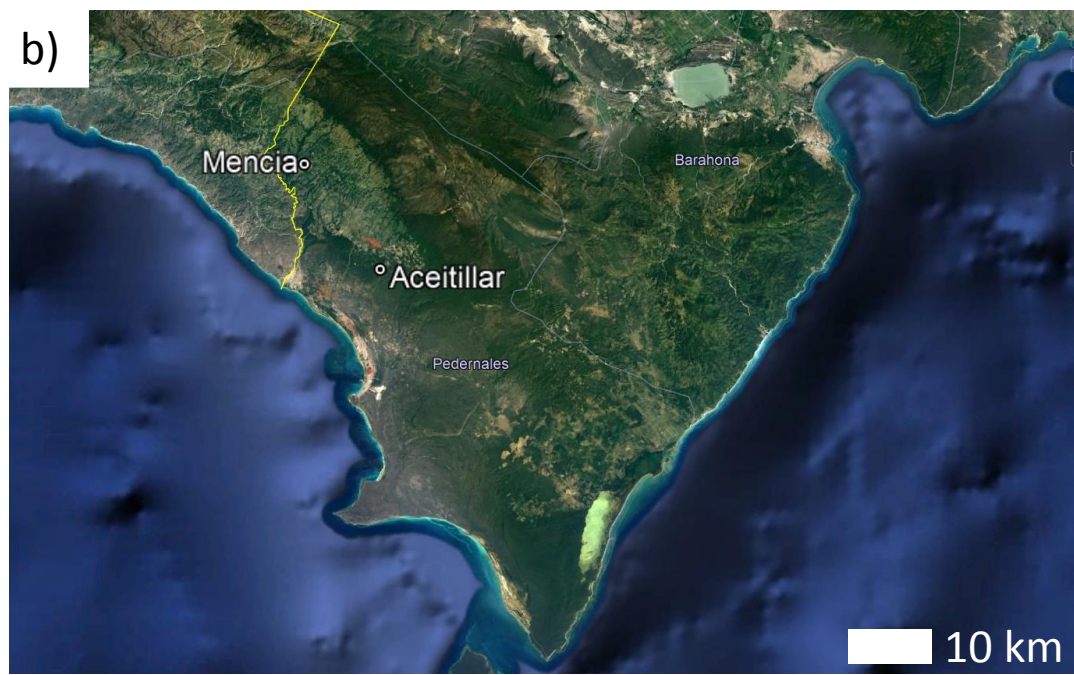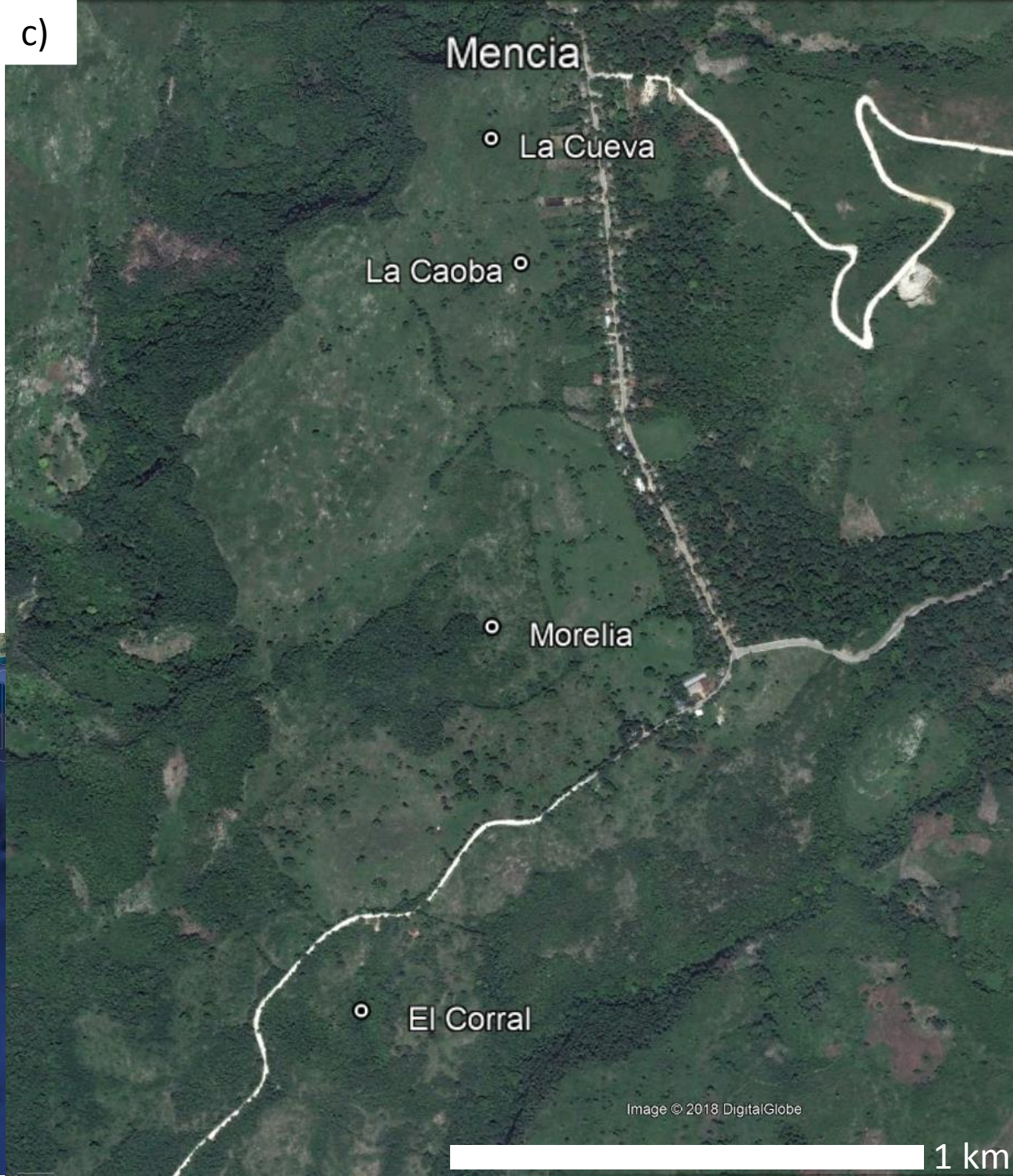

Supplement: Appendix S1 — (a) The Dominican Republic with locations of the four Mencia pasture sites and Aceitillar broadleaf reference site; (b) the Pedernales region; (c) location of four study sites near Mencia, Dominican Republic including La Cueva (abandoned for 2 yr when the study began), La Caoba (5 yr), Morelia (10 yr), and El Corral (20 yr). [file peerj-06-5217-s001.pdf]
