# Supplementary material for: Avian community characteristics and demographics reveal how conservation value of regenerating tropical dry forest changes with forest age
Source: PeerJ. 2018 Jul 10;6:e5217. doi: 10.7717/peerj.5217 (PMC6044266; doi:10.7717/peerj.5217)
Supplement: Appendix S2 [file peerj-06-5217-s002.docx]

**Supplemental Information, Appendix S2**

**Temporal and age relationships for a chronosequence of four abandoned pastures and a mature dry forest reference site near Mencia, Dominican Republic**.

All abandoned pastures were studied from the winter of 2002-2003 through 2007-2008. The reference site at Aceitillar was studied from the winter of 1996-1997 through 2001-2002. Summaries of vegetation characteristics are listed above the ages the sites were observed.

|  | **Mean  canopy** | | Grasses, forbs and woody shrubs. Few shrubs or trees. Most vegetation < 1.5 m | | | | | | | | Most vegetation < 4 m. High shrub diversity, some trees | | | | |  | Shrub density / diversity decreases while canopy height and cover increase. | | | | |  | Closed-canopy dry forest (reference site) | | | | | |
| --- | --- | --- | --- | --- | --- | --- | --- | --- | --- | --- | --- | --- | --- | --- | --- | --- | --- | --- | --- | --- | --- | --- | --- | --- | --- | --- | --- | --- |
|  |  |  | **Year since pasture was abandoned** | | | | | | | | | | | | | | | | | | | | | | | | | |
|  | **Ht^1^** | **Cov^2^** | **2** | **3** | **4** | **5** | **6** | **7** | **8** | **9** | **10** | **11** | **12** | **13** | **14** | **//** | **20** | **21** | **22** | **23** | **24** | **//** | **Mature dry forest** | | | | | |
| **La Cueva** | 3.6 | 18 | 2003 -2004 through  2007-2008 | | | | |  |  |  |  |  |  |  |  |  |  |  |  |  |  |  |  |  |  |  |  |  |
| **La Caoba** | 3.9 | 22 |  |  |  | 2003 -2004 through  2007-2008 | | | | |  |  |  |  |  |  |  |  |  |  |  |  |  |  |  |  |  |  |
| **Morelia** | 5.2 | 54 |  |  |  |  |  |  |  |  | 2003 -2004 through  2007-2008 | | | | |  |  |  |  |  |  |  |  |  |  |  |  |  |
| **El Corral** | 6.4 | 76 |  |  |  |  |  |  |  |  |  |  |  |  |  |  | 2003 -2004 through  2007-2008 | | | | |  |  |  |  |  |  |  |
|  |  |  |  |  |  |  |  |  |  |  |  |  |  |  |  |  |  |  |  |  |  |  |  |  |  |  |  |  |
| **Aceitillar** | 10.6 | 94 |  |  |  |  |  |  |  |  |  |  |  |  |  |  |  |  |  |  |  |  | 1996 -1997 through  2001-2002 | | | | | |

^1^Ht = Mean canopy height

^2^Cov = Mean canopy cover
