# Supplementary material for: Avian community characteristics and demographics reveal how conservation value of regenerating tropical dry forest changes with forest age
Source: PeerJ. 2018 Jul 10;6:e5217. doi: 10.7717/peerj.5217 (PMC6044266; doi:10.7717/peerj.5217)
Supplement: Appendix S3 [file peerj-06-5217-s003.docx]

**Supplemental Information, Appendix S3**

**Results of Generalized Linear Mixed Model (GLMM) analysis of change in bird community and population characters across a chronosequence of four abandoned pastures and a mature dry forest reference site near Mencia, Dominican Republic**.

Regression analyses used time as a continuous variable for the four pasture sites over the five years they were studied. 1-way ANOVA analyses used site as an ordered categorical variable and used linear and quadratic trend tests to examine change across the entire chronosquence. P values < 0.05 are in bold.

|  |  | **Regression** | |  |  |  | **1-way ANOVA** | |  |  |  | |  | |  |  |
| --- | --- | --- | --- | --- | --- | --- | --- | --- | --- | --- | --- | --- | --- | --- | --- | --- |
|  |  |  |  |  |  |  | **Linear trend** | |  |  | **Quadratic trend** | | | |  |  |
|  | **Variable** | **Slope** | **(SE)** | **χ^2^** | **df** | **P** | **Effect size** | **(SE)** | **Z** | **P** | **Effect size** | **(SE)** | | **Z** | | **P** |
| **Community-level data** | **Species richness** | -0.02 | 0.01 | 5.7 | 4,5 | 0.02 | -0.99 | 0.41 | -2.4 | **0.016** | -0.18 | 0.47 | | -0.39 | | 0.69 |
|  | **Simpson's diversity** | -0.18 | 0.08 | 9.6 | 4,5 | 0.002 | -14.7 | 2.63 | -5.6 | **<0.001** | -8.73 | 2.86 | | -3.05 | | **0.00** |
|  | **Evenness** | 0.024 | 0.02 | 4.2 | 4,5 | 0.04 | -0.7 | 0.46 | -1.5 | 0.1287 | -1.17 | 0.49 | | -2.40 | | **0.02** |
|  |  |  |  |  |  |  |  |  |  |  |  |  | |  | |  |
| **Population Sex Ratio** | **Migrants** |  |  |  |  |  | 2.08 | 0.91 | 2.3 | **0.022** | 1.27 | 1.02 | | 1.25 | | 0.21 |
|  | **Residents** |  |  |  |  |  | 0.45 | 0.94 | -0.5 | 0.63 | -0.17 | 1.05 | | -0.16 | | 0.87 |
