# Supplementary material for: Avian community characteristics and demographics reveal how conservation value of regenerating tropical dry forest changes with forest age
Source: PeerJ. 2018 Jul 10;6:e5217. doi: 10.7717/peerj.5217 (PMC6044266; doi:10.7717/peerj.5217)
Supplement: Appendix S4 [file peerj-06-5217-s004.docx]

**Supplemental Information, Appendix S4**

**Results of test of 1-way ANOVA-style models for linear and quadratic trends in the abundance of individual Neotropical migrant species.**

Means and approximate 95% confidence intervals for each site are given in terms of captures per 1000 net hour. Means were calculated from the GLMM used to fit the model and 95% CIs calculated as ±1.96*SE.

OVEN = Ovenbird, BAWW = Black and White Warbler, AMRE = American Redstart, BTBW = Black Throated Blue Warbler, PAWA = Palm Warbler, PRAW = Prairie Warble.
