# Supplementary material for: Avian community characteristics and demographics reveal how conservation value of regenerating tropical dry forest changes with forest age
Source: PeerJ. 2018 Jul 10;6:e5217. doi: 10.7717/peerj.5217 (PMC6044266; doi:10.7717/peerj.5217)
Supplement: Appendix S5 [file peerj-06-5217-s005.docx]

**Supplemental Information, Appendix S5**

**Modeled capture rates from 1-way ANOVA style generalized linear mixed models for migrant species at four pasture sites and one reference forest (Aceitillar).**

Sites are ordered along x-axis in order of successional stage. Error bars are approximate 95% confidence intervals. See Supplemental Materials Table S2 for test statistics for tests for linear and quadratic trends.


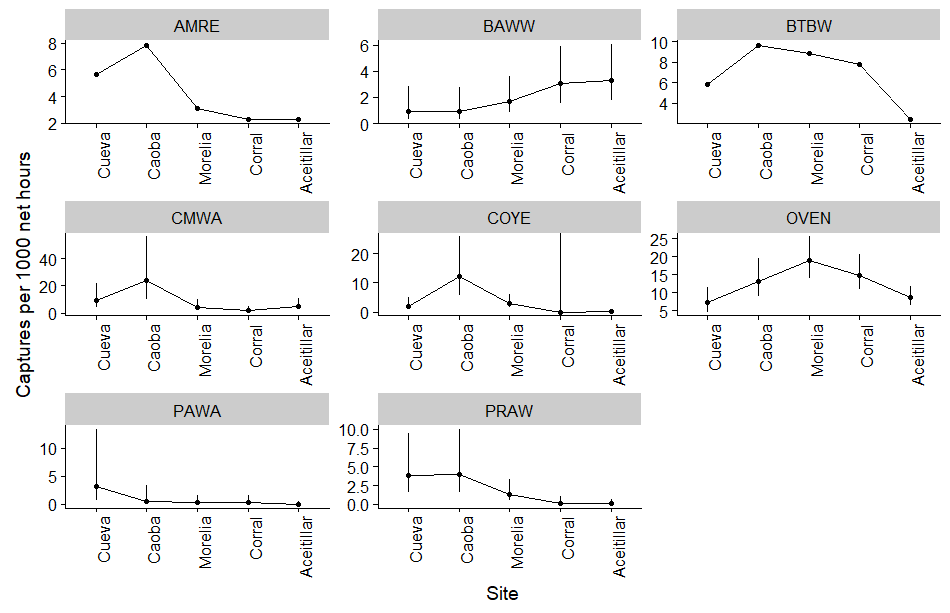


AMRE = American Redstart, BAWW = Black and White Warbler, BTBW = Black-Throated Blue Warbler, CMWA = Cape May Warbler, COYE = Common Yellowthroat, OVEN = Ovenbird, PAWA = Palm Warbler, PRAW = Prairie Warbler
