# Supplementary material for: Avian community characteristics and demographics reveal how conservation value of regenerating tropical dry forest changes with forest age
Source: PeerJ. 2018 Jul 10;6:e5217. doi: 10.7717/peerj.5217 (PMC6044266; doi:10.7717/peerj.5217)
Supplement: Appendix S6 [file peerj-06-5217-s006.docx]

**Supplemental Information, Appendix S6**

**Significance of “pasture age^2^“ terms in regression-type generalized linear mixed models (GLMM) examining non-linear patterns in bird abundance.**

Peak Abundance = Year when mean abundance was highest. “.” Indicates p < 0.10, “*” indicates p < 0.05, and “**” p < 0.01.

OVEN = Ovenbird, BAWW = Black and White Warbler, COYE = Common Yellow Throat, AMRE = American Redstart, CMWA = Cape May Warbler, BTBW = Black-throated Blue Warbler, PAWA = Palm Warbler, PRAW = Prairie Warbler, MAWA = Magnolia Warbler.
