# Supplementary material for: Avian community characteristics and demographics reveal how conservation value of regenerating tropical dry forest changes with forest age
Source: PeerJ. 2018 Jul 10;6:e5217. doi: 10.7717/peerj.5217 (PMC6044266; doi:10.7717/peerj.5217)
Supplement: Appendix S7 [file peerj-06-5217-s007.docx]

**Supplemental Information, Appendix S7**

**Demographics of overwintering Neotropical migrants, residents, and endemics in regenerating broadleaf habitat of different ages, and in mature dry forest in the Sierra de Bahoruco, Dominican Republic.**

Data pooled for each site and pooled across all years. Tallies only include first instance of an individual being captured each year. Individuals recaptured in multiple years count toward each years’ totals. N = total number of individual captures, and n = number of individuals used to calculate the proportion of after-hatch year individuals (AHY) or proportion male. Scientific names found in Table 1.

|  |  | **La Cueva** | | |  | **La Caoba** | | |  | **Morelia** | | |  | **El Corral** | | |  | **Aceitillar** | | |
| --- | --- | --- | --- | --- | --- | --- | --- | --- | --- | --- | --- | --- | --- | --- | --- | --- | --- | --- | --- | --- |
|  |  | **(2-6 years)** | | |  | **(5-9 years)** | | |  | **(10-14 years)** | | |  | **(20-24 years)** | | |  | **Mature forest reference** | | |
| **Residency** | **Species** | **N** | **AHY (n)** | **Male (n)** |  | **N** | **AHY (n)** | **Male (n)** |  | **N** | **AHY (n)** | **Male (n)** |  | **N** | **AHY (n)** | **M (n)** |  | **N** | **AHY (n)** | **Male (n)** |
| Neo- tropical migrant | OVEN | 79 | 0.53 (79) |  |  | 85 | 0.49 (85) | 0 (1) |  | 141 | 0.5 (141) | 0 (4) |  | 103 | 0.41 (103) |  |  | 140 | 0.62 (140) |  |
|  | BAWW | 7 | 0.71 (7) | 0.43 (7) |  | 7 | 0.43 (7) | 0.43 (7) |  | 14 | 0.21 (14) | 0.36 (14) |  | 23 | 0.39 (23) | 0.26 (23) |  | 77 | 0.48 (77) | 0.48 (77) |
|  | COYE | 19 | 0.58 (19) | 0.63 (19) |  | 110 | 0.57 (110) | 0.58 (107) |  | 24 | 0.58 (24) | 0.42 (24) |  | 0 |  |  |  | 1 | 0 (1) | 1 (1) |
|  | AMRE | 49 | 0.67 (49) | 0.2 (41) |  | 54 | 0.5 (54) | 0.22 (46) |  | 26 | 0.73 (26) | 0.19 (16) |  | 18 | 0.39 (18) | 0.36 (14) |  | 43 | 0.49 (43) | 0.35 (43) |
|  | CMWA | 90 | 0.52 (90) | 0.34 (90) |  | 173 | 0.55 (173) | 0.4 (173) |  | 42 | 0.5 (42) | 0.36 (39) |  | 15 | 0.2 (15) | 0.67 (15) |  | 96 | 0.59 (96) | 0.58 (96) |
|  | BTBW | 59 | 0.56 (59) | 0.41 (59) |  | 59 | 0.58 (59) | 0.42 (59) |  | 68 | 0.63 (68) | 0.49 (68) |  | 55 | 0.53 (55) | 0.45 (55) |  | 49 | 0.65 (49) | 0.53 (49) |
|  | PAWA | 23 | 0.43 (23) |  |  | 13 | 0.46 (13) |  |  | 3 | 0 (3) |  |  | 3 | 0.33 (3) |  |  | 1 | 1 (1) |  |
|  | PRAW | 27 | 0.63 (27) | 0.59 (17) |  | 37 | 0.57 (37) | 0.31 (29) |  | 11 | 0.64 (11) | 0.56 (9) |  | 1 | 0 (1) | 0 (1) |  | 3 | 0.67 (3) | 0.67 (3) |
| Resident | STOF | 23 | 0.36 (11) |  |  | 9 | 0.33 (3) |  |  | 21 | 0.75 (4) |  |  | 17 | 0.75 (4) |  |  | 60 | 0 (25) |  |
|  | RLTH | 56 | 0.28 (54) |  |  | 58 | 0.22 (58) |  |  | 22 | 0.23 (22) |  |  | 18 | 0.44 (18) |  |  | 66 | 0 (16) |  |
|  | NOMO | 31 | 0.13 (31) |  |  | 34 | 0.18 (34) |  |  | 20 | 0.05 (20) |  |  | 19 | 0.42 (19) |  |  | 3 | 0 (3) |  |
|  | BANA | 121 | 0.34 (121) |  |  | 90 | 0.33 (90) | 1 (1) |  | 55 | 0.27 (55) |  |  | 22 | 0.32 (22) |  |  | 100 | 0.12 (100) |  |
|  | YFGR | 175 | 0.55 (175) | 0.7 (151) |  | 114 | 0.55 (113) | 0.68 (99) |  | 61 | 0.47 (60) | 0.65 (52) |  | 54 | 0.56 (54) | 0.63 (51) |  | 2 | 0 (1) | 1 (2) |
|  | BFGR | 26 | 0.46 (26) | 0.35 (26) |  | 41 | 0.37 (41) | 0.49 (35) |  | 12 | 0.67 (12) | 0.42 (12) |  | 8 | 0.38 (8) | 0.38 (8) |  | 2 | 0 (1) | 0.5 (2) |
|  | GABU | 102 | 0.63 (102) | 0.64 (74) |  | 128 | 0.57 (128) | 0.53 (99) |  | 78 | 0.68 (78) | 0.57 (44) |  | 56 | 0.43 (56) | 0.71 (42) |  | 379 | 0.51 (376) | 0.52 (241) |
| Endemic | HILC | 16 | 0.13 (15) |  |  | 15 | 0.14 (14) |  |  | 16 | 0 (15) |  |  | 27 | 0.07 (27) |  |  | 10 | 0 (1) |  |
|  | GTGT | 30 | 0.57 (30) |  |  | 59 | 0.41 (59) |  |  | 19 | 0.37 (19) |  |  | 40 | 0.38 (40) |  |  | 128 | 0.23 (124) |  |
|  | BCPT | 68 | 0.55 (67) |  |  | 60 | 0.62 (60) |  |  | 59 | 0.47 (59) |  |  | 70 | 0.5 (70) |  |  | 139 | 0.53 (129) |  |

Neotropical migrants: OVEN = Ovenbird, BAWW = Black-and-white Warbler, COYE = Common Yellowthroat, AMRE = American Redstart, CMWA = Cape May Warbler, BTBW = Black -throated Blue Warbler, PAWA = Palm Warbler, PRAW = Prairie Warbler.

Residents: STOF = Stolid Flycatcher, RLTH = Red-legged Thrush, NOMO = Northern Mockingbird, BANA = Bananaquit, YFGR = Yellow-faced Grassquit, BFGR = Black-faced Grassquit, GABU = Greater Antillean Bullfinch.

Endemics: HILC = Hispaniolan Lizard-Cuckoo, GTGT = Green-tailed Ground-Tanager, BCPT = Black-crowned Palm-Tanager
