# Supplementary material for: Avian community characteristics and demographics reveal how conservation value of regenerating tropical dry forest changes with forest age
Source: PeerJ. 2018 Jul 10;6:e5217. doi: 10.7717/peerj.5217 (PMC6044266; doi:10.7717/peerj.5217)
Supplement: Appendix S8 [file peerj-06-5217-s008.docx]

**Supplemental Information, Appendix S8**

**Percentage of captured individuals (n) of 10 resident species that were site persistent in regenerating pastures and mature dry forest in the Sierra de Bahoruco, Dominican Republic.**

Site persistence is determined by mist net recaptures, and/or resighting of individuals which were uniquely color-banded (CB). Tallies only include first instance of an individual being captured each year. Individuals recaptured in multipole years count toward each year’s totals. Scientific names of species are found in Table 1.

Neotropical migrants: OVEN = Ovenbird, BAWW = Black-and-white Warbler, COYE = Common Yellowthroat, AMRE = American Redstart, CMWA = Cape May Warbler, BTBW = Black -throated Blue Warbler, PAWA = Palm Warbler, PRAW = Prairie Warbler.

Residents: STOF = Stolid Flycatcher, RLTH = Red-legged Thrush, NOMO = Northern Mockingbird, BANA = Bananaquit, YFGR = Yellow-faced Grassquit, BFGR = Black-faced Grassquit, GABU = Greater Antillean Bullfinch.

Endemics: HILC = Hispaniolan Lizard-Cuckoo, GTGT = Green-tailed Ground-Tanager, BCPT = Black-crowned Palm-Tanager
