# Supplementary material for: Avian community characteristics and demographics reveal how conservation value of regenerating tropical dry forest changes with forest age
Source: PeerJ. 2018 Jul 10;6:e5217. doi: 10.7717/peerj.5217 (PMC6044266; doi:10.7717/peerj.5217)
Supplement: Appendix S9 [file peerj-06-5217-s009.docx]

**Supplemental Information, Appendix S9**

**Percentage composition of flying insects captured on sticky traps (top) and terrestrial insects in leaf litter samples (bottom) from four abandoned pasture sites and a mature forest reference.**


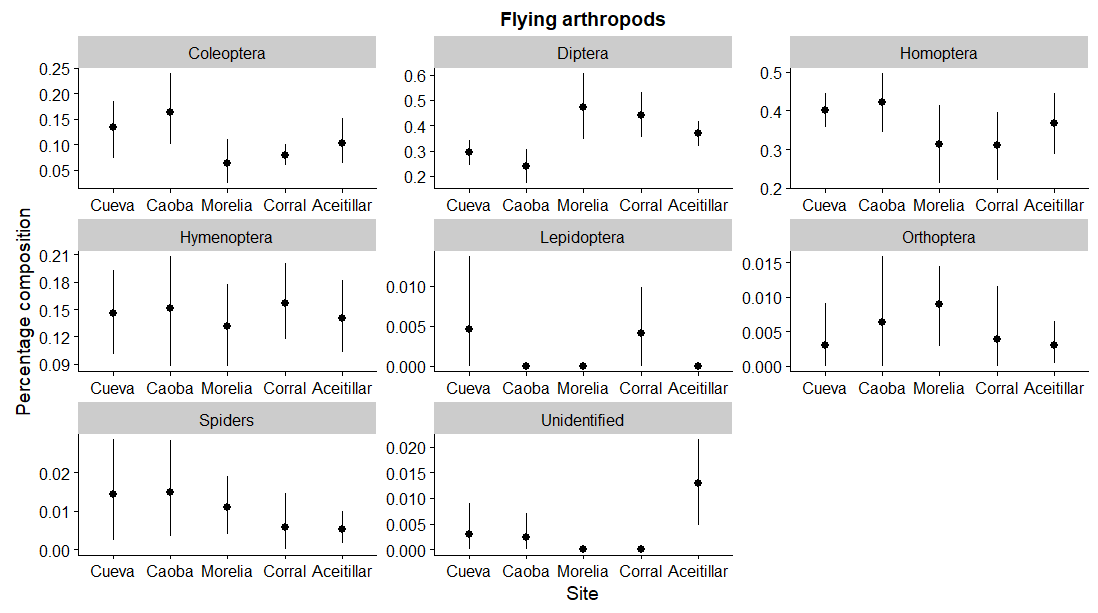


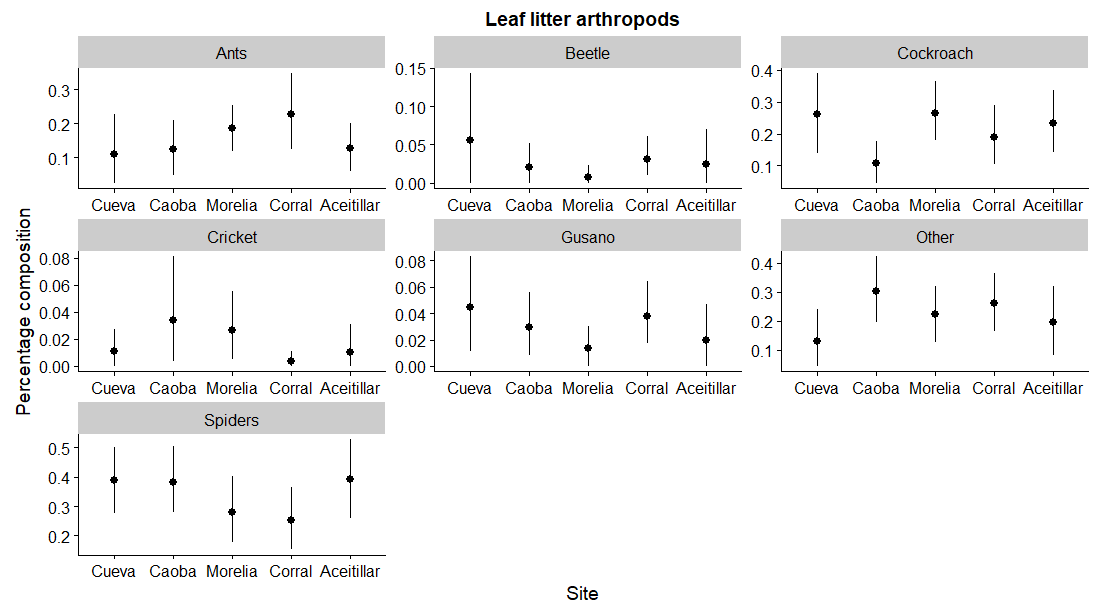


Flying arthropods:

**(323) (348) (599) (399) (749)**

**La Cueva La Caoba Morelia El Corral Aceitillar**

**Leaf-litter arthropods**

**(210) (227) (213) (294) (112)**

**La Cueva La Caoba Morelia El Corral Aceitillar**
