# Supplementary material for: Avian community characteristics and demographics reveal how conservation value of regenerating tropical dry forest changes with forest age
Source: PeerJ. 2018 Jul 10;6:e5217. doi: 10.7717/peerj.5217 (PMC6044266; doi:10.7717/peerj.5217)
Supplement: Appendix S10 [file peerj-06-5217-s010.docx]

**Supplemental Information, Appendix S10**

**Results of linear and quadratic trend tests of scaled mass index (SMI) from 1-way ANOVA style linear models for migrant and resident species at four pasture sites and one reference forest (Aceitillar).**

Sites are ordered along x-axis in order of successional stage. Error bars are approximate 95% confidence intervals. See Supplemental Materials Table S3for test statistics for tests for linear and quadratic trends.

|  | **Species** | **AMRE** | **BAWW** | **BTBW** | **CMWA** | **OVEN** | **BANA** | **BCPT** | **GABU** | **GTGT** | **STOF** |
| --- | --- | --- | --- | --- | --- | --- | --- | --- | --- | --- | --- |
| **Linear trend** | **β** | 0.472 | 0.750 | 0.141 | 0.088 | -0.398 | -0.648 | -2.399 | -2.664 | -0.823 | 0.254 |
|  | **t** | 1.390 | 0.927 | 0.391 | 0.192 | -0.760 | -2.646 | -3.399 | -3.407 | -2.320 | 0.307 |
|  | **p** | 0.168 | 0.357 | 0.697 | 0.848 | 0.448 | **0.009** | **0.001** | **0.001** | **0.021** | 0.760 |
|  |  |  |  |  |  |  |  |  |  |  |  |
| **Quadratic trend** | **β** | -0.287 | 0.525 | -0.447 | 0.818 | 0.327 | -0.768 | -0.166 | -2.315 | -0.893 | -0.321 |
|  | **t** | -0.599 | 0.573 | -1.108 | 1.367 | 0.538 | -2.508 | -0.185 | -2.279 | -1.701 | -0.330 |
|  | **p** | 0.551 | 0.569 | 0.270 | 0.173 | 0.591 | **0.013** | 0.853 | **0.023** | **0.091** | 0.742 |
|  |  |  |  |  |  |  |  |  |  |  |  |
|  | **La Cueva** | 7.1 | 9.6 | 8.8 | 9.4 | 18.5 | 7.5 | 27.6 | 21.2 | 12.4 | 18.9 |
|  | **La Caoba** | 7.1 | 9.1 | 8.8 | 9.3 | 18.6 | 7.6 | 27.2 | 21.5 | 12.3 | 18.9 |
| **Means** | **Morelia** | 7.2 | 9.4 | 8.9 | 9.3 | 18.3 | 7.7 | 26.9 | 20.9 | 12.4 | 19.1 |
|  | **El Corral** | 7.3 | 10.0 | 8.9 | 9.1 | 18.3 | 7.4 | 27.2 | 21.3 | 12.5 | 19.0 |
|  | **Aceitillar** | 7.2 | 9.5 | 8.8 | 9.5 | 18.4 | 7.3 | 26.4 | 19.9 | 11.9 | 19.0 |

AMRE = American Redstart, BAWW = Black and White Warbler, BTBW = Black-Throated Blue Warbler, CMWA = Cape May Warbler, OVEN = Ovenbird, BANA = Banaquit, BCPT = Black-Crowned Palm Tanager, GRWA = Green Warbler, STOF = Stolid Flycatcher
