# Supplementary material for: Avian community characteristics and demographics reveal how conservation value of regenerating tropical dry forest changes with forest age
Source: PeerJ. 2018 Jul 10;6:e5217. doi: 10.7717/peerj.5217 (PMC6044266; doi:10.7717/peerj.5217)
Supplement: Appendix S11 [file peerj-06-5217-s011.docx]

**Supplemental Information, Appendix S11**

**Modeled mean scaled mass index (SMI) from 1-way ANOVA style linear models for migrant species at four pasture sites and one reference forest (Aceitillar).**

Sites are ordered along x-axis in order of successional stage. Error bars are approximate 95% confidence intervals. See Supplemental Materials Table S4 for test statistics for tests for linear and quadratic trends.


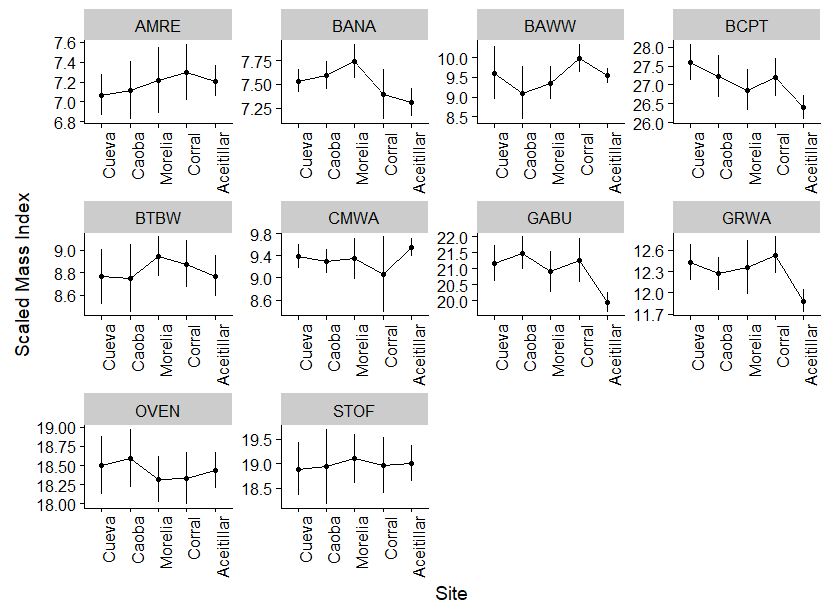


AMRE = American Redstart, BAWW = Black and White Warbler, BTBW = Black-Throated Blue Warbler, CMWA = Cape May Warbler, OVEN = Ovenbird, BANA = Banaquit, BCPT = Black Crowned Palm Tanager, GRWA = Green Warbler, STOF = Stolid Flycatcher
